# Association between colic and sleep problems in infancy and subsequent development, emotional and behavioral problems: a longitudinal study

**DOI:** 10.1186/s12887-020-02483-1

**Published:** 2021-01-07

**Authors:** Lisbeth Valla, Milada Cvancarova Småstuen, Randi Andenæs, Nina Misvær, Christine Olbjørn, Sølvi Helseth

**Affiliations:** 1 Department of Nursing and Health promotion,Faculty of Health Sciences, Oslo Metropolitan University, Oslo, Norway; 2 Department of Pediatric and Adolescent Medicine, Akershus University Hospital, Lørenskog, Norway; 3 Institute of Clinical Medicine, Campus Ahus, University of Oslo, Oslo, Norway

**Keywords:** Sleep, Colic, Infancy, Development, The Norwegian mother, Father and child cohort study

## Abstract

**Background:**

Sleep and colic problems in infancy have been linked to adverse health outcome, but there is limited knowledge of the association between sleep and colic problems in infancy and subsequent development, emotional and behavior problems in young children. The aim of the present study was to examine whether there is an associations between infants’ crying and sleep problems at 6 months and behavioral and development problems at 18 months, 3 and 5 years.

**Methods:**

This study is based on the Norwegian Mother, Father and Child Cohort Study (MoBa), conducted at the Norwegian Institute of Public Health from June 1999 to December 2008. A total of 86,724 children were included. Colic and sleep (sleep duration, nocturnal awakenings and easy to put to bed) was assessed by mother-reports. Z-scores were used to assess differences between groups of children (e.g. having colic or not, having a sleep problem or not). Emotional and behavioral problems were measured with items from the Child Behavior Checklist. Development problems were measured with items from The Ages and Stages Questionnaire.

**Results:**

Infants with colic scored significantly lower on development at 5 years (B=-0.10, CI [− 0.14 to - 0.06]) and higher on internalizing problems both at 3 years (B=0.15. CI [0.11 to 0.18]) and 5 years (B=0.17. CI [0.12 to 0.21]) than the reference population. Children who awoke frequently and were more difficult to put to bed at 6 months scored significantly lower on development at 18 months and 3 and 5 years, and higher on internalizing behavior problems at 3 and 5 years (B=0.18 and B=0.16). Children with shorter sleep duration at 6 months had more internalizing behavior problems at 3 years (B=0.14. CI [0.07 to 0.21]) and 5 years (B=0.15. CI [0.05 to 0.25]) than the reference population.

**Conclusions:**

Colic and sleep problems early in life should be taken into account as risk factors for development and behavioral problems within the first 5 years of a child’s life.

**Supplementary Information:**

The online version contains supplementary material available at 10.1186/s12887-020-02483-1.

## Background

Crying and sleep problems in infancy are common concerns for parents [1, 2], often resulting in increased use of multiple health services [3–5]. Sleep problems have different presentations and rates at different ages [2], and the prevalence varies depending on the criteria used. Between 10 and 35% of parents report problems with their infant’s sleep, including both sleep duration and nighttime awakenings [1, 6–8]. A crying duration exceeding 3 h/24 h for at least 3 days of at least 3 weeks is typically known as ‘colic’ [9]. Infantile colic affects between 17 and 25% of infants during the first months of life, with a reported peak of excessive crying somewhere between 3 and 6 weeks of age [10, 11].

Although infant crying and sleep problems are usually regarded as self-resolving [10], there is conflicting evidence on their association with adverse medium to long-term outcomes. In a meta-analysis [12], both excessive crying and sleep problems during the first year of life were linked to internalizing and general behavioral problems in children aged 2 to 10 years, particularly in multi-problem families. These findings were confirmed in a recent systematic review that found that sleep duration among healthy children aged 0–4 years, based on parents’ reports, is prospectively associated with mental health problems such as anxiety, depression, and poor emotion regulation. The evidence related to cognitive and motor development was not clear [13]. Two previous Norwegian birth cohort studies have examined sleep in young children [14, 15], and found that total sleep, prolonged sleep onset, and frequent nighttime awakening among 24-month-old children was associated with a greater risk of concurrent social-emotional problems in toddlerhood [14]. Children who sleep less than 11 h or awaken three or more times per night at 18 months have more emotional regulation difficulties at 5 years [15].

Although previous research represents important steps toward identifying the long-term outcome of colic and sleep problems in children, these studies have limitations. Most previous studies have examined the associations in preschool children rather than in the first years of life. Due to high neuroplasticity and rapid brain development in the first years of life, early experiences can modify the organization of cortical structures and strengthen the brain connections [16]. Hence, understanding the relationship between difficulties already in infancy will enable researchers and clinicians to identify, prevent, and treat sleep and crying problems.

The Norwegian Mother and Child Cohort Study (MoBa) contains valuable information on infant crying and sleep behavior and children’s health and development from birth to toddlerhood. Thus, the aim of the present study was to examine whether there is an associations between infants’ crying and sleep problems at 6 months and behavioral and development problems at 18 months, 3 and 5 years.

## Methods

### Study population

#### The Norwegian mother and child cohort study

The present study uses data from The Norwegian Mother and Child Cohort study (MoBa), a prospective population- based cohort study conducted by the Norwegian Institute of Public Health from 1999 through 2008 [17]. The MoBa is a study of health and causes of disease among mothers and children. Pregnant women were invited to take part in the study around the time of the ultrasound examination in week 17–20 of gestation. All pregnant women in Norway were eligible for participation in MoBa if they were able to read Norwegian. Each woman could participate with more than one pregnancy. About 41% of the invited women consented to participate. The MoBa cohort included 114.500 children and 95.200 mothers. In MoBa biological material from mother and child, as well as questionnaire data have been collected since pregnancy and until the child is 8 years old. Questionnaires are completed by the mothers at given time points in the follow up (at gestational week 15, gestational week 30, and when the child was 6 months and 18 months, and 3, 5, and 8 years), and linked to national health registries. Participation rates were about 92.2% at week 22 in pregnancy, 84.8% at 6 months, 72.5% at 18 months, 58.5% at 3 years and 53.0% at 5 years [17].

The current study is based on version 10 of the MoBa data files. The establishment of MoBa and initial data collection was based on a license from the Norwegian Data Protection Agency and approval from The Regional Committees for Medical and Health Research Ethics. The MoBa cohort is now based on regulations related to the Norwegian Health Registry Act. The current study was approved by The Regional Committees for Medical and Health Research Ethics (project no; 2017/2199).

For the present study, we have analyzed data on 86,724 children. Twins (*n*= 1480) and triplets (*n*= 14) were excluded. Data was collected at four time points, when the child was 6 months (Questionnaire 4), 18 months (Questionnaire 5), 3 years (Questionnaire 6) and 5 years (5 years Questionnaire).

### Measures

#### Demographic variables

Demographic variables included child gender, gestational age, maternal age, maternal education level, civil and work status.

### Emotional and behavioral problems (CBCL)

The child’s emotional, social and behavioral problems was assessed with the Child Behavior Checklist (CBCL) [18]. The CBCL/1.5–5 consists of 99 items. The items rates as not true (0 points), somewhat or sometimes true (1points), or very true or often true (2 points). There are several syndrome scales in the CBCL, and the scales are designated as Emotionally Reactive, Anxious/Depressed, Somatic Complaints, Withdrawn, Attention Problems, and Aggressive Behavior [18]. The title of each scale summarizes the kinds of problems that form the syndrome. In addition to the syndrome scales, the CBCL/1.5–5 can be scored in terms of two broad groupings of syndromes, internalizing (Emotion/Reactive, Anxious/Depressed, Somatic Complaints,) and externalizing behavior problems (Attention Problems, Aggressive Behavior) [18]. The CBCL has been proven to be a valid and reliable tool [19]. Due to space restrictions in the MoBa questionnaires, an abbreviated version of the full CBCL scales were used, including 19 items at 18 months, 26 items at 3 years and 27 items at 5 years. The MoBa abbreviated externalizing scale are previous found to be representative of the full externalizing CBCL scale [20] In the present study, the CBCL was collected at 18 months, 3 years and 5 years.

### The ages and stages questionnaire (ASQ II)

The child’s general development was assessed with the ASQ [21]. The ASQ, 2nd edition, consists of 19 age-specific questionnaires intended for use from the age of 4 to 60 months old. Each questionnaire in the ASQ consists of 30 items covering five developmental areas: communication, gross motor, fine motor, problem solving, and personal-social development The ASQ items rates yes (10 points), sometimes (5 points), or not yet (0 points). The ASQ has shown good test–retest reliability (94%), and concurrent validity when compared to standardized tests (76–88%) [21]. The Norwegian version of the ASQ has also shown good validity [22]. In the MoBa study, an abbreviated ASQ scale was used, including a 13-item scale at 18 months, a 10-item scale at 3 years and a 12-item scale at 5 years, respectively. In the present study, the ASQ was collected at 18 months, 3 years and 5 years.

### Sleep-related variables

Child sleep was measured by single questions about the child’s quantity of sleep “How many hours does your child sleep per day?”, nighttime awakenings “How often does the child wake up during the night?” and about how easy the child is to put to bed at night “Is the child easy to put to bed and falls asleep fast? ” [17]. The response options for sleep duration were categorized as follows: 0: sleep duration were less than 8 h,1: 8–10 h, 2: 11–12 h, 3: 13 to 14 h, 4: 14 h or more of sleep. For statistical analyses, the variable was further operationalized as appropriate sleep > 10 h per day vs not-appropriate sleep. The response options for nighttime awakenings were as follows: 0: 3 or more times every night, 1: once or twice every night, 2: a few times a week, 3: seldom or never. Further, in a similar way to sleep duration, this variable was dichotomized for statistical analyses as not frequent vs frequent awakenings. The reference category was not frequent defined as twice every night or more seldom (values 1 or higher of the original variable). The question about how easy the child is to put to bed was dichotomized as follows: easy to put to bed and not easy to put to bed.

### Colic

Colic was assessed by the parent reporting on a question when her child was 6 months: ‘Has the child had colic?. The response categories were yes or no. Parents thus answered yes if they thought their child had had colic, and no other diagnostic criteria were needed.

## Statistical analyses

Continuous variables were described with mean and standard deviation (SD), categorical variables with counts and percentages. Both main outcomes were assessed using a questionnaire administered at four time points. The number of included items varied among assessment points so we used z-scores to be able to assess differences between groups of children both at given time points and across the whole follow-up. The z-scores were constructed as follows: at each assessment point the dataset was divided into two groups based on the exposure variable (e.g. having colic or not, having a sleep problem or not). Those who did not report such an event (no colic, no sleep problems) served as the normal population for a given outcome (event). Thus z-scores equal to zero represent the mean for the normal population, scores over zero indicate higher values of the outcome compared to the norm, and scores below zero indicate values lower than 50% of the norm (Supplementary file 1).

To model changes at given time points and across the follow-up trajectories, we used linear mixed models for repeated measures with an unstructured covariance matrix to account for dependencies within the included individuals (children), as the same child was assessed at several time points. The results are expressed as estimates of beta (B) with 95% confidence intervals (CI). *P*-values < 0.05 were considered statistically significant. As the study is considered exploratory, no correction for multiple testing was done. All analyses were performed using SPSS, version 24.

## Results

The participants’ characteristics are described in Table 1. There were 51.0% boys in the sample, and a large proportion of mothers had a medium to high level of education and were married or co-habiting.

**Table 1:** Characteristics of the study population when the child is 6 months

	n (%)	mean (SD)	Median (range)
**Child (*N*= 86,724) mean (SD)**			
Male **n (%)**	40, 205 (51.0)		
Missing (*n*= 7947)			
Gestation age			
≥37 weeks	70,815 (95.0)		
32–36 weeks	3231 (4.3)		
28–31 weeks	309 (0.4)		
< 28 weeks	192 (0.3)		
Missing (*n*= 12,177)			
Birth weight in grams	78,816		3600 (500, 5960)
Missing (*n* = 7908)			
**Mother (N= 86,724)**			
Maternal age	82,377	29.8 (4.5)	
Missing (*n* = 4387)			
Maternal education ^a^			
low	26,607 (34.0)		
medium	32,941 (42.0)		
high	18,836 (24.0)		
Missing (*n* = 8340)			
Work status ^b^			
Education	4930 (6.0)		
Employed	68,153 (83.1)		
Other	8938 (10.9)		
Missing (*n* = 4703)			
Civil status ^c^			
Married/co-habiting	80,333 (97.9)		
Missing (*n* = 4640)			

^a^ Education at 16–20 weeks of pregnancy: low education = completed elementary school and or high school. Medium education = completed college / university 1–3 years. High education = completed college / university 4 years

^b^ Work Status at 16–20 weeks of pregnancy: education = student, at home, trainee, military service. Employed = employed in the public or private sector. Other = unemployed

^c^ Civil status at 16–20 weeks of pregnancy

### ASQ questionnaire

When the whole follow-up was considered, our data for ASQ revealed small but statistically significant differences between children with colic and disruptive sleep early in life and the reference population. Firstly, we assessed possible differences over time between children who had colic and children who did not have colic. Compared to baseline, ASQ scores were slightly but statistically significantly lower at 5 years (B=-0.10, CI [-0.14 to - 0.06]) for children with colic. With respect to sleep duration, when comparing children who did not have enough sleep at baseline to children for whom enough sleep was reported, we found significantly higher scores for ASQ at 18 months (B=0.09, CI [0.04 to 0.14]) and 3 years (B=0.08, CI [0.01 to 0.14]) but there were no difference in ASQ scores between the children at 5 years. However, it has to be kept in mind that the differences at 18 months and 3 years, although statistically significant, were very small. When we compared children who awoke frequently at night to those who did not at baseline, there were small but statistically significant differences between the children concerning the ASQ scores at 18 months (B=-0.06, CI [-0.01 to - 0.02) and 3 years (B=-0.01, CI [-0.01 to - 0.03]). Lastly, we compared children who were easy to put to bed to those who were difficult in that respect. Children for whom the parents reported difficulties in falling asleep scored significantly lower on ASQ compared to those who were easy to put to bed, at 18 months (B=-0.06, CI [-0.08 to - 0.03) and also at 5 years ( B=-0.09, CI [-0.01 to - 0.05]) (Table 2).

**Table 2:** Mixed effects analysis of the relationship between ASQ and colic and sleep problems

	ASQ		
Variable	B	95% CI	***p***-values
**Colic**			
Time			
Baseline (6 months)	ref		
18 months	− 0.03	− 0.06 to 0.00	0.02
3 years	0.03	− 0.00 to - 0.06	0.07
5 years	−0.10	− 0.14 to - 0.06	< 0.01
**Sleep duration** ^a^			
Time			
Baseline (6 months)	ref		
18 months	0.09	0.04 to 0.14	< 0.01
3 years	0.08	0.01 to 0.14	0.02
5 years	−0.02	− 0.11 to 0.07	0.62
**Frequent nocturnal awakenings** ^b^			
Time			
Baseline (6 months)	ref		
18 months	−0.06	−0.01 to - 0.02	< 0.01
3 years	−0.01	−0.01 to - 0.03	< 0.01
5 years	0.01	−0.01 to - 0.02	0.26
**Easy to put to bed** ^**c**^			
Time			
Baseline (6 months)	ref		
18 months	−0.06	−0.08 to − 0.03	< 0.01
3 years	0.03	0.00 to − 0.5	0.04
5 years	−0.09	− 0.01 to − 0.05	< 0.01

*Adjusted for ICQ. Mother’s education, mother’s age

^a^Sleep duration: Hours of sleep per day

^b^Frequent nocturnal awakenings: Usually wakes during the night

^c^Easy to put to bed: Easy to put to bed and falls asleep quickly

### CBCL questionnaire

As shown in Table 3, children with colic at baseline compared to children who did not have colic scored significantly higher on CBCL internalizing problems both at 3 years ( B=0.15. CI [0.11 to 0.18]) and 5 years ( B=0.17. CI [0.12 to 0.21]) and significantly lower on CBCL externalizing problems.

**Table 3:** Mixed effects analysis of the relationship between CBCL and colic and sleep problems

	CBCL		
Variable	B	95% CI	***p***-values
Colic			
Internalizing			
Time			
Baseline (18 months)	ref		
3 years	0.15	0.11 to 0.18	< 0.01
5 years	0.17	0.12 to 0.21	< 0.01
Externalizing			
Time			
Baseline (18 months)	ref		
3 years	−0.06	−0.09 to − 0.03	< 0.01
5 years	− 0.05	- 0.08 to − 0.01	< 0.01
Sleep duration^a^			
Internalizing			
Time			
Baseline (18 months)	ref		
3 years	0.14	0.07 to 0.21	< 0.01
5 years	0.15	0.05 to 0.25	< 0.01
Externalizing			
Time			
Baseline (18 months)	ref		
3 years	−0.21	−0.28 to −0.15	< 0.01
5 years	−0.11	− 0.18 to 0.04	< 0.01
Frequent nocturnal awakenings ^b^			
Internalizing			
Time			
Baseline (18 months)	ref		
3 years	0.06	0.05 to 0.07	< 0.01
5 years	0.06	0.04 to 0.08	< 0.01
Externalizing			
Time			
Baseline (18 months)			
3 years	−0.09	−0.10 to −0.08	< 0.01
5 years	−0.09	− 0.11 to − 0.09	< 0.01
Easy to put to bed ^c^			
Internalizing			
Time			
Baseline (18 months)	ref		
3 years	0.18	0.15 to 0.21	< 0.01
5 years	0.16	0.12 to 0.20	< 0.01
Externalizing			
Time			
Baseline (18 months)	ref		
3 years	−0.18	−0.21 to −0.16	< 0.01
5 years	−0.19	− 0.22 to − 0.16	< 0.01

*Adjusted for ICQ. Mother’s education, mother’s age

^a^Sleep duration: Hours of sleep per day

^b^Frequent nocturnal awakenings: Usually wakes during the night

^c^Easy to put to bed: Easy to put to bed and falls asleep quickly

Children who slept too little at baseline scored significantly higher on internalizing problems both at 3 years (B=0.14. CI [0.07 to 0.21]) and 5 years (B=0.15. CI [0.05 to 0.25]) compared to children who had enough sleep at baseline and significantly lower on CBCL externalizing problems. Children whose parents reported that the child awoke frequently at night scored significantly higher on CBCL internalizing problems and significantly lower on CBCL externalizing problems compared to children who did not awake frequently at night at baseline. Children who were not easy to put to bed at baseline compared to children who were easy in that respect scored significantly higher on CBCL internalizing problems both at 3 and 5 years (B=0.18 and B=0.16) respectively. They also scored 18% lower on externalizing problems at 3 years and similarly 19% lower at 5 years compared to the mean scores of those who were easy to put to bed.

## Discussion

This large population-based study revealed that infants with colic scored significantly lower on general development at 5 years and higher on internalizing problems both at 3 and 5 years than the reference population. Children who awoke frequently and were more difficult to put to bed at 6 months scored significantly lower on general development at 18 months and 3 and 5 years, and higher on internalizing behavior problems at 3 and 5 years than the reference population. Children with shorter sleep duration at 6 months had more internalizing behavior problems at 3 and 5 years.

Our results are consistent with the findings of previous research [12, 13, 15, 23], which concluded that colic and sleep problems early in life may predict later development and behavioral difficulties. In accordance with previous studies [15, 24–26], our findings revealed that internalizing behavioral problems are most frequently reported in association with sleep problems in young children. In a previous Norwegian cohort study, Sivertsen and colleagues (2015) found that shorter sleep duration at 18 months was predictive of developing emotional regulation difficulties utilizing the CBCL both concurrently and at 5 years of age, especially in relation to internalizing problems. However, our study extends previous findings by showing that children with shorter sleep duration already from 6 months of age had more internalizing behavior problems at later ages than their peers. Behaviour difficulties identified in young children can persist and increase a child’s risk of later adverse outcomes, although stability and pathways of behavior problems seems to vary according to the types of problem that are studied [27, 28].

Our results also revealed that children in the group with frequent awakenings or difficulty with putting to bed had poorer general developmental outcomes at preschool ages than children with no such problems. One explanation for our findings may be that frequent awakenings predict poorer attention regulation [29, 30] and affect executive task performance that is important for the ability to learn new skills and solve problems in everyday life [31, 32]. Although difficulties falling asleep or frequent awakenings in children are often followed by longer sleeps on subsequent days or nights, these results highlight the importance of nighttime sleep as a component of total sleep duration and are consistent with the literature supporting a positive association between nighttime sleep duration and development outcome in preschool children [23].

Current empirical knowledge on the development of excessive criers after the end of the difficult colic period is inconclusive. Our results showed that infants with colic reported at 6 months of age scored significantly lower on general development and higher on internalizing problems then the reference population. These findings are confirmed by previous studies, which reported an association between infant crying and subsequent child behavioral problems [12, 33], especially in externalizing behaviors and lower motor scores [34]. Conversely, Bell et al. (2018) did not find any associations in an Australian study between infants with colic whose crying self-resolved and internalizing and externalizing behavior problems measured by caregiver reports [35]. However, some of the infants with colic in our study may have had multiple regulatory problems (crying combined with sleep problems) and thus may have been at greater risk of developmental deficits later in life compared with infants with isolated crying problems [12]. In addition, we did not have any information about crying after 6 months which may affect long-term development.

Different underlying mechanisms have been proposed to explain the predictive links between early regulation problems and subsequent development problems, including overlapping genetic and biomedical features [36] and parenting factors [37]. Sleep disturbance may influence the brain circuits that underlie emotional processing that are key characteristics of emotional and behavioral problems [38, 39]. Children with individual differences in personality, temperament, behavior, and social competence trigger positive or negative reactions from parents. When parents are affected by their infants’ fragmented sleep or crying, they may provide less optimal stimulation overall, inadequate parental practices or limit-setting behavior that may negatively affect development and behavior [37].

This study showed that infants with colic and sleep problems at 6 months scored significantly lower on general development, and higher on behavior problems than the comparison group, for example, children with colic at 6 months scored 10% lower on developmental problems at 5 years compared to the mean scores of children without colic, and children with shorter sleep duration scored 15% higher on internalizing problems at 3 years compared to the mean scores of children with longer sleep duration. The clinical relevance of this findings should be further investigated in later research. However, previous research revealed that crying and sleeping problems in infancy predict later development and behavior difficulties [12, 33, 40].

Our findings extend the outcomes to a younger age group and underscore the importance of preventive measures and supporting families with infants with colic and sleep problems. Several risk and protective factors for sleep problems have been identified [41], and identification of problems and support for families and appropriate referral are needed throughout infancy and early childhood.

Although the present study has many strengths, including the large prospective longitudinal sample with repeated follow-ups, the results must be interpreted in light of several methodological limitations. Firstly, the assessment of sleep or colic did not include validated or objective measures and mother-reported sleep duration was assessed in predefined categories. Secondly, we did not include all the items in the ASQ or CBCL instrument, and thus the use of abbreviated versions instead of the original scales may have affected the results. Thirdly, some confounders were controlled for in this study, while other variables that could have impacted the analyzed associations, such as maternal psychopathology or maternal burden of care were left unexplored. Lastly, the results may also be affected by a selection bias due to high attrition rates in the MoBa study [17]. When comparing MoBa participants with the data from the Medical Birth Registry in Norway (including all women giving birth in Norway) on key parameters, a lower rate on predictor variables was found, with higher maternal age and fewer health related risks, than children of those not participating. Still, we would advise caution when interpreting our findings.

## Conclusion

This large population-based study revealed that colic and disruptive sleep early in life may be risk factors for developmental and behavioral problems within the first 5 years of a child’s life. It is important to be aware that disruptive sleep and colic in infancy may have long-term negative consequences. Thus, early intervention should be a part of a comprehensive plan to prevent behavioral and developmental difficulties.

## Supplementary Information

**Additional file 1.**

**Additional file 2.**

**Additional file 3.**

**Additional file 4.**

**Additional file 5.**

## Data Availability

The data that support the findings of this study are available from Norwegian Institute of Public Health, but restrictions apply to the availability of these data, which were used under license for the current study, and so are not publicly available. Data are however available from the author upon reasonable request and with permission of the Norwegian Institute of Public Health.

## Abbreviations

MoBa: The Norwegian Mother and Child Cohort Study
ASQ: Ages and Stages Questionnaires
CBCL: The Child Behavior Checklist
