# Supplementary material for: Association between colic and sleep problems in infancy and subsequent development, emotional and behavioral problems: a longitudinal study
Source: BMC Pediatr. 2021 Jan 7;21:23. doi: 10.1186/s12887-020-02483-1 (PMC7788887; doi:10.1186/s12887-020-02483-1)
Supplement: Supplementary file 1 — Additional file 1. [file 12887_2020_2483_MOESM1_ESM.docx]

|  | **Colic** | | **Easy to put to bed** | | **Frequent nocturnal awakenings** | | **Sleep duration** | |
| --- | --- | --- | --- | --- | --- | --- | --- | --- |
| **Time** | **Mean** | **SD** | **Mean** | **SD** | **Mean** | **SD** | **Mean** | **SD** |
| 6 months | 0.071 | 1.055 | 0.038 | 1.038 | -0.063 | 0.925 | -0.032 | 1.058 |
| 18 months | 0.036 | 1.043 | -0.014 | 0.989 | -0.121 | 0.855 | 0.080 | 1.155 |
| 3 years | 0.093 | 1.090 | 0.064 | 1.065 | -0.045 | 0.938 | 0.039 | 1.174 |
| 5 years | -0.050 | 1.083 | -0.066 | 1.130 | -0.069 | 1.069 | -0.065 | 1.266 |

Supplementary table 1: The z-scores for children with colic, easy to put to bed, frequent nocturnal awakenings and sleep duration, respectively calculated using children without those conditions as the comparison group. (the z-scores for the comparison groups are always mean=0, SD=1).
