# Supplementary material for: Association between colic and sleep problems in infancy and subsequent development, emotional and behavioral problems: a longitudinal study
Source: BMC Pediatr. 2021 Jan 7;21:23. doi: 10.1186/s12887-020-02483-1 (PMC7788887; doi:10.1186/s12887-020-02483-1)
Supplement: Supplementary file 2 — Additional file 2. [file 12887_2020_2483_MOESM2_ESM.pdf]

Den norske **mor- og barn**-undersøkelsen

Questionnaire before starting school – at approximately 5 years old

The questionnaire will be processed by a computer. It is therefore important to us use a blue or black ballpoint pen and write clearly.

- In the small boxes you should put a cross in the box that is most relevant like this: ☒
- If you think that you have put a cross in the wrong box, correct it by filling in the box completely like this: ■

Specify the day, month and year when the questionnaire was completed ☐☐ day ☐☐ month ☐☐☐☐ year *(write the year in full, e.g. 2010)*

About the child

1. What is your child's height and weight nowadays?

Height ☐☐☐ cm Weight ☐☐,☐ kg

Date of measurement ☐☐ month ☐☐☐☐ year

2. Who do you live with?

- ☐ Spouse
- ☐ Coihabitant
- ☐ Other adults
- ☐ Children of others
- ☐ None

3. If children lives with you, how many and what ages?
(Also include the child you are filling out this form for)

Number of children 5 years old or older ☐☐

Number of children 3 or 4 years old ☐☐

Number of children from 0 to 3 years old ☐☐

4. Do you live with the child's father?

☐ Yes ☐ No ☐ Have never lived with the child's father

If NO, how old was the child when you separated/ moved apart ☐,☐ år

Childcare

5. Where is the child looked after in the daytime these days?
(You may tick several boxes)

	No. hours per week
☐ Nanny/ au pair/ outdoor nursery	☐☐
☐ Family kindergarten	☐☐
☐ Private kindergarten	☐☐
☐ Public kindergarten	☐☐
☐ Family members other than mother/father	☐☐

6. If your child is attending kindergarten, is it organized in traditional units or as bases/large groups?

- ☐ Unit-kindergarten
- ☐ Base-kindergarten

7. If the child is looked after another place than home, how many adults are looking after the child (e.g number of adults in the unit/base)?

☐☐ adults

8. How many other children are cared for in the same child care? (If kindergarten, state the number of children in the same unit/base) ☐☐ children

9. How many times has the child changed child care? (Do not include change of unit within the kindergarten) ☐ times

10. How old was the child when he/she started in current child care? ☐☐ months

11. Does your child receive, or has received any extra resources in the kindergarten?

☐ No ☐ Yes Number of hours per week ☐☐

12. How does your child like being in the current child care?

Not at all	Not much	Both likes and dislikes	Mostly	Very much
☐	☐	☐	☐	☐

5b år Moba 7.000 09.2011 WJ

13. If your child is looked after some other place than at home or with a child minder, to what extent are you satisfied with different aspect of the child care? *(Cross off one response to each statment, from dissatisfied to very satisfied)*

	Dissatisfied	More dissatisfied than satisfied	Both satisfied and dissatisfied	More satisfied than dissatisfied	Very satisfied
1. Types of activities in the kindergarten	☐	☐	☐	☐	☐
2. The way the kindergarten prepare activities of importance for starting school	☐	☐	☐	☐	☐
3. The expertise of the kindergarten staff for doing a goodjob with your child	☐	☐	☐	☐	☐
4. The food served in the kindergarten (healthy, appropriate nutritional meals)	☐	☐	☐	☐	☐
5. Information about how your child is doing	☐	☐	☐	☐	☐

Media and games

14. Does your child have a TV in his/her own room? ☐ NO ☐ Yes

**15. How many hours does the child watch TV/DVD or play PC/TV-games?**	Never	Less than 1 hour	From 1 up to 3 hours	From 3 up to 5 hours	5 hours or more
1. On a typical weekday	☐	☐	☐	☐	☐
2. On a typical day during the weekend	☐	☐	☐	☐	☐

**16. How often does the child use...**	Daily	4-6 days a week	2-3 days a week	1 day a week	Never/ rarely
1. PC/ computer at home?	☐	☐	☐	☐	☐
2. TV-games/ handheld electronic games (e.g. Playstation, Game Boy, PSP)?	☐	☐	☐	☐	☐
3. PC /TV games where the purpose is educational (learn things that are relevant for school)?	☐	☐	☐	☐	☐
4. Books as an activity and entertainment?	☐	☐	☐	☐	☐
5. Drawing/painting etc.as activity and entertainment?	☐	☐	☐	☐	☐

Child development and illnesses

17. The following questions concern any illnesses or health problems you child has had. Has your child ever suffered, or is currently suffering from any of the following long-term illnesses or health problems?

			If yes, was the illness/problem confirmed by a doctor/psychologist?		If yes, does the child still have the illness/problem?	
	No	Yes	No	Yes	No	Yes
1. Asthma	☐	☐	☐	☐	☐	☐
2. Pollen allergy/hayfever	☐	☐	☐	☐	☐	☐
3. Obstructed/wheesing in chest	☐	☐	☐	☐	☐	☐
4. Epilepsy	☐	☐	☐	☐	☐	☐
5. Cerebral palsy	☐	☐	☐	☐	☐	☐
6. Impaired hearing	☐	☐	☐	☐	☐	☐
7. Delayed motor development or clumsy	☐	☐	☐	☐	☐	☐
8. Delayed or deviant language development	☐	☐	☐	☐	☐	☐
9. Unusually restless/hyperactive/ADHD	☐	☐	☐	☐	☐	☐
10. Attention problems/difficulties concentrating	☐	☐	☐	☐	☐	☐
11. Autism/autistic traits	☐	☐	☐	☐	☐	☐
12. Asperger syndrome	☐	☐	☐	☐	☐	☐
13. Behavioral problems (difficult and unruly)	☐	☐	☐	☐	☐	☐
14. Emotional difficulties (sad and worried)	☐	☐	☐	☐	☐	☐
15. Impaired vision (patch treatment/need for daily use of glasses)	☐	☐	☐	☐	☐	☐
16. Other, specify________________			☐	☐	☐	☐

18. More about the childs health

+

	No	Yes	
1. Has the child had an injury, resulting in a diagnosis?................................	☐	☐	Describe: ______
2. Does the child have a learning disability or mental development delay?	☐	☐	Describe: ______
3. Does the child have a syndrome or suspected of having a syndrome?.....	☐	☐	Describe: ______
4. Has the child had other serious, but **short term** illnesses?.......................	☐	☐	Describe: ______
5. Has the child ever been a witness to close familiy being subject to violence?	☐	☐	Describe: ______

+

19. Developmental milestones

	No	Yes
1. Did your child say his/her first words before 2 years of age (do not include mum and dad)?	☐	☐
2. Did your child start combining words before 2,5 years of age (combine 2-3 words into sentences)?	☐	☐
3. Did your child stop using diapers in the daytime before 4 years of age (tick yes if less than 3 accidents per month)?	☐	☐

20. Has a professional ever assessed your child as having reduced hearing?

☐ No ☐ Yes

If yes, at what age? (Enter a cross in several boxes if necessary)

☐ Before 18 months
☐ 18 - 36 months
☐ later than 36 months

+

21. Has your child been referred to the following services?

	No	Yes
Habilitation services	☐	☐
Child psychiatric clinic/ department	☐	☐
Educational psychology services	☐	☐

If yes, what was the reason for the referral?

______

22. Has your child been assessed for language delay or other difficulties with language/speech or communication?

☐ No ☐ Yes

If yes: What was the conclusion after the assessment?
(You may enter several crosses)

1. Everything was fine, no difficulties.................................. ☐
2. Only delay in spoken language, good language comprehension.. ☐
3. Delay in both using spoken language and ability to understand spoken language.. ☐
4. Difficulties in pronunciation .... ☐
5. Stammer or stutters when talking .... ☐
6. Other language issues .... ☐

Describe: ______

______

23. Has anyone in the child's close family ever had any of the following problems? *(Only include the child's biological relatives)*

+

We are especially interested in the child's siblings, parents, grandparents, uncles, aunts or cousins.

	No	Yes	
1. Been a late talker as a child................................	☐	☐	If Yes, specify the relationship: ______
2. Had difficulties learning to read and write...........	☐	☐	If Yes, specify the relationship: ______
3. Had difficulties in pronouncing sounds as a child (preschool)...	☐	☐	If Yes, specify the relationship: ______

+

24. About the child's pronounciation

(Enter a cross in a box from 1-5 with 1 being very difficult and 5 being very easy.)

	Very difficult 1	2	Varies 3	4	Very easy 5
1. How easy it is for you to understand what your child's speech?	☐	☐	☐	☐	☐
2. How easy it is for strangers to understand what your child's speech?	☐	☐	☐	☐	☐

Food supplements and eating habits

25. Is your child taking any of the following dietary supplements? *(Enter a cross in a box for each line, for both frequency and amount and fill in brand name.)*

	Numer of times per week					Amount per time		
**Liquid dietary supplements**	No	6-7	4-5	1-3	<1	1 tsp	1 csp	1 ss
Cod liver oil	☐	☐	☐	☐	☐	☐	☐	☐
Omega 3, brand name: ______	☐	☐	☐	☐	☐	☐	☐	☐
Sanasol/Biovit	☐	☐	☐	☐	☐	☐	☐	☐
Other liquid dietary supplement, brand name: ______	☐	☐	☐	☐	☐	☐	☐	☐

	Times a week					Amount per time		
**Capsules/tablets**	No	6-7	4-5	1-3	<1	1	2	3+
Omega 3, brand name: ______	☐	☐	☐	☐	☐	☐	☐	☐
Cod liver oil	☐	☐	☐	☐	☐	☐	☐	☐
Multivitamines, brand name: ______	☐	☐	☐	☐	☐	☐	☐	☐
Fluoride tablets	☐	☐	☐	☐	☐	☐	☐	☐
Other dietary supplements, brand name: ______	☐	☐	☐	☐	☐	☐	☐	☐

	Rarely/ never	Once a week	2-3 times per week	4-6 times per week	Every day
**26. How often does your child eat breakfast (at home or in the kindergarten)?**	☐	☐	☐	☐	☐

27. Is the following correct for your child for the last 6 months?

	No	Yes
1. Has your child ever eaten what most people would consider a really large amount of food?	☐	☐
2. Have you ever had the impression that your child could not stop eating or that he/she could not control what or how much he/she was eating?	☐	☐

	Twice a week or more	Once a week	More rarely	Never
3. How often has your child been eating a really large amount of food where you at the same time had the impression that the child did not have control?	☐	☐	☐	☐

Language and preschool activities

ASQ

28. The child's ability to understand and tell

Here are some questions about children's oral language andwhat they understand. Maybe your child already has done some of the activities described here, and some the child has not started doing yet. Tick the box for each question you find suitable for your child.

	Yes	Some-times	Not yet
1. Can the child tell you at least two thing about a familiar object? If you f.ex.. say: "Tell me about the ball" can the child answer something like "It is round and I can throw it and it is big"	☐	☐	☐
2. Without giving your child help by pointing or repeating directions, does your child follow three direction that are unrelated to one another? Give all three directions before your child starts. For example, you may ask your child to "Clap your hands, walk to the door and sit down" or "Give me the pen, open the book and stand up"	☐	☐	☐
3. Does your child use four- and five- word sentences? For example, does your child say, "I want the car"?	☐	☐	☐
4. When talking about something that already happened, does your child use word that end in "ed" such as walked, jumped or played? Ask your child questions such as "How did you get to the store?" ("We walked") "What did you do at your friends house?" ("We played").	☐	☐	☐
5. Does you child use comparison words such as heavier, stronger or shorter? Ask your child questions, such as "A car is big, but a bus is _____" (bigger"); "A cat is heavy, but a man is ____" (heavier); A TV is small, but a book is ____ " (smaller).	☐	☐	☐
6. Does your child answer the following questions:1. "What do you do when you are hungry?" (Acceptable answers include: "Get food", "Eat", " Ask for something to eat", and "Have a snack".) 2. "What do you do when you are tired?" (Acceptable answers include: "Take a nap", "Rest", "Go to sleep", "Go to bed", "Lie down", and "Sit down").	☐	☐	☐
7. Does your child repeat the sentences shown below back to you, without any mistakes? You may repeat each sentence one time. Mark "yes" if your child repeats both sentences without mistakes or "sometimes" if your child repeats one sentence without mistakes. "Jane hides her shoes for Maria to find" - "Al read the blue book under his bed"."	☐	☐	☐

Ages and Stages Questionnaires, 2nd Ed., Bricker et al. ©1999 Paul H. Brookes Publishing Co. Norsk utgave ©2003 R.BUP Kopiering forbudt ifølge lov.

29. What is the mother tongue of the child's mother and father and what language(s) does the child speak?

	Mother's mother tongue	Father's father tongue	What language(s) does the child speak? *(you may enter several crosses)*
1. Norwegian, Danish or Swedish	☐	☐	☐
2. Other Nordic languages (Icelandic, Finish) or Sami	☐	☐	☐
3. Western European languages (for example German, English, Spanish).	☐	☐	☐
4. Other languages (Eastern European, Asian, Turkish, African)	☐	☐	☐

30. About the child's language experiences.

	Only Norwegian	More Norwegian than other language	As much Norwegian as other language	More other language than Norwegian	Only other language
1. What language(s) do you speak with your child?	☐	☐	☐	☐	☐
2. What language(s) does your spouse/partner speak with your child?	☐	☐	☐	☐	☐
3. What language(s) does the child speak with his/her siblings?	☐	☐	☐	☐	☐

31. Factors of importance for language skills.

Relatiely to other children of the same age, to which degree does the following questions serve to dercribe the child's language performance? Use the scale from 1 to 5 to express your view.

	Quite wrong 1	2	Both yes and no 3	4	Quite right 5
1. Forgets words she/he knows the meaning of	☐	☐	☐	☐	☐
2. Mixes up words with similar meaning	☐	☐	☐	☐	☐
3. Has difficulties in understanding the meaning of common words	☐	☐	☐	☐	☐
4. Has difficulties in responding to questions just as quickly as others	☐	☐	☐	☐	☐
5. Is often searching for the right words	☐	☐	☐	☐	☐
6. Has difficulties in using complete sentences	☐	☐	☐	☐	☐
7. Is using short sentences when s/he is responding to questions	☐	☐	☐	☐	☐
8. Has difficulties in retelling a story s/he has heard	☐	☐	☐	☐	☐
9. Is quickly getting tired in tasks demanding attention to language	☐	☐	☐	☐	☐
10. It doesn't seem like what s/he is learning is remembered	☐	☐	☐	☐	☐
11. Has difficulties in remembering things	☐	☐	☐	☐	☐
12. Difficulties in understands what others are saying	☐	☐	☐	☐	☐
13. Misconceive instructions and when told to	☐	☐	☐	☐	☐
14. Has problems with remembering messages	☐	☐	☐	☐	☐
15. Misunderstands context and what is going on	☐	☐	☐	☐	☐
16. Is difficult to understand	☐	☐	☐	☐	☐
17. Has difficulties in expressing wishes and needs	☐	☐	☐	☐	☐
18. Is not understood by others	☐	☐	☐	☐	☐
19. Is not initiating communication and are active in use of language	☐	☐	☐	☐	☐
20. Has difficulties in pronunciation	☐	☐	☐	☐	☐
21. Is able to have a dialogue with peers	☐	☐	☐	☐	☐
22. Avoids talking to other people than close family	☐	☐	☐	☐	☐

32. About the child's language competence.

How typical is the statement for your child:

	Rarely or never	Some-times	Regulary	Often or always
1. It is hard to make sense of what he/ she is saying, even though the words are clearly spoken.	☐	☐	☐	☐
2. Gets the sequence of events muddled up when trying to tell a story or describe a recent event. E.g., if describing a film, might talk about the end before the begining.	☐	☐	☐	☐
3. Uses terms like "he" or "it" without making it clear what he/she is talking about. For instance, when talking about a film, might say "he was really great" without explaining who "he" is.	☐	☐	☐	☐
4. Talks clearly about what he/she plans to do in the future (e.g. what he/she will do tomorrow, or plans for going on holiday).	☐	☐	☐	☐
5. Can be hard to tell if he/ she is talking about something real or make-believe.	☐	☐	☐	☐
6. Explains a past event clearly (e.g. what he/she did at school, or what happened at a football game).	☐	☐	☐	☐

Children's Communication Checklist- Second Edition (CCC-2).
Copyright © 2003 Pearson, Assessment. Used with permission. All rights reserved.

	No	Yes
7. Does the child talk about things that is going to happen in the near future, like the weekend, e.g. " Tomorrow, we'll go to the movies".	☐	☐
8. Does the child talk about things that has already happened, e.g. "Yesterday, we took the bus to kindergarten"	☐	☐
9. Does the child talk about things that could or can happen, e.g." If he touches the stove top, he could burn himself"	☐	☐
10. Does the child talk in a special way when pretending to be someone else, e.g. "Now you were the king and I was the queen".	☐	☐

33. About the child's pre-school activities

	Very poor/ poor	Average	Good/ very good
1. How would you rate your child's ability to tell a story?	☐	☐	☐
2. How would you rate your child's ability to communicate his/ her own needs in a way understandable to adults and friends?	☐	☐	☐

During a typical week:

	Never	Seldom	Sometimes	Often	Very often
3. How often do you teach your child how to print letters and words?	☐	☐	☐	☐	☐
4. How often do you help your child read letters and sounds?	☐	☐	☐	☐	☐

	Nei	Ja
5. Would you say that your child is interested in writing letters?	☐	☐
6. Would you say that your child is generally interested in books?	☐	☐
7. Would you say that your child is able to read simple words?	☐	☐
8. Would you say that your child is able to read simple sentences?	☐	☐
9. Would you say that your child is able to write his/ her name?	☐	☐

10. About how many minutes does your child like to sit still when you read for him/her?

Does not like it at all	☐
Less than 5 minutes	☐
6-15 minutes	☐
16-45 minutes	☐
More than 45 minutes	☐
Will not be read to	☐

Child's skills and behavior

34. Child's play

The following scale examines various behaviors that children may engage in during indoor free play. Although it is true that childrens' behaviors may be quite variable, please try to make a general evaluation of the child's 'everyday' behavior.

	Never	Hardly ever	Some-times	Often	Very often
1. Talks to other children during play	☐	☐	☐	☐	☐
2. Plays by himself/herself, examining an object or toy	☐	☐	☐	☐	☐
3. Plays 'rough-and tumble' with other children	☐	☐	☐	☐	☐
4. Takes on the role of onlooker or spectator	☐	☐	☐	☐	☐
5. Plays 'make-believe' with other children	☐	☐	☐	☐	☐
6. Engages in group play	☐	☐	☐	☐	☐
7. Engages in pretend play by hilself/herself	☐	☐	☐	☐	☐
8. Plays alone, building things with blocks and/or other toys	☐	☐	☐	☐	☐
9. Wanders around aimlessly	☐	☐	☐	☐	☐
10. Plays in goups with (not just beside) other children	☐	☐	☐	☐	☐
11. Plays 'make-believe' but not with other children	☐	☐	☐	☐	☐
12. Watches, or listens to other children without trying to join in	☐	☐	☐	☐	☐
13. Engages in playful/mock fighting with other children	☐	☐	☐	☐	☐
14. Plays by himself/herself, drawing, painting pictures or doing puzzles	☐	☐	☐	☐	☐
15. Engages in active conversations with other children during play	☐	☐	☐	☐	☐
16. Engages in pretend play with other children	☐	☐	☐	☐	☐
17. Plays alone, exploring toys or objects, trying to figure out how they work	☐	☐	☐	☐	☐
18. Remains alone and unoccupied, perhaps staring off into space	☐	☐	☐	☐	☐
19. Plays by him/herself, engaging in simple motor activities (e.g. running)	☐	☐	☐	☐	☐
20. Plays just for a short while with each toy, does not settle with any toy	☐	☐	☐	☐	☐

35. Activities and restlessness

Please rate each item according to your child's behavior in the last month..

	Not true at all	Just a little true	Pretty much true	Very much true
1. Inattentive, easily distracted	☐	☐	☐	☐
2. Short attention span	☐	☐	☐	☐
3. Fidgets with hands or feet or squirms in seat	☐	☐	☐	☐
4. Messy or disorganized at home or in the kindergarten	☐	☐	☐	☐
5. Only attends if it is something he/she is very interested in	☐	☐	☐	☐
6. Distractibility or attention span a problem	☐	☐	☐	☐
7. Avoids, expresses reluctance about, or has difficulties engaging in tasks that require sustained mental effort (such as activities in kindergarten or helping out at home)	☐	☐	☐	☐

	Not true at all	Just a little true	Pretty much true	Very much true
8. Gets distracted when given instructions to do something	☐	☐	☐	☐
9. Has trouble concentrating in kindergarten	☐	☐	☐	☐
10. Leaves seat in kindergarten or in other situations in which remaining seated is expected	☐	☐	☐	☐
11. Does not follow through on instructions and fails to finish tasks in kindergarten, chores or duties at home (not due to oppositional behavior or failure to understand instrucions)	☐	☐	☐	☐
12. Easily frustrated in efforts	☐	☐	☐	☐

36. About motor skills

Enter a cross for each line if your child masters these activities.

	No	Yes
1. Do you think your child walks, runs, and climbs like other children at the same age?	☐	☐
2. Able to stand on one foot for at least 5 sec without problems keeping balance	☐	☐
3. Hops, on one foot , many times, without support	☐	☐
4. Plays "catch" with other children; throwing to him/her and catching the ball at least half the time	☐	☐
5. Swings on a swing, pumping by self	☐	☐
6. Rides a two-wheeled bike, with or without training wheels	☐	☐
7. Puts together a puzzle with nine or more pieces	☐	☐
8. Draws or copies a square with straight corners	☐	☐
9. Cuts with scissors, following a simple outline or pattern	☐	☐
10. Draw pictures of complete people that have at least head: with eyes-nose-mouth; body. Arms and legs, hands and feet (need to do all seven for a yes)	☐	☐
11. Coloures withing the lines in a colouring book	☐	☐
12. Does your child show interest in and likes to participate in sports or active games requiring good motor skills?	☐	☐

37. About temperament and personal style

How typical are the following statements for your child's behavior? *(Enter a cross in a box for each line)*

	Very typical	Quite typical	Neither/ nor	Not so typical	Not at all typical
1. Your child is always on the go	☐	☐	☐	☐	☐
2. Your child is off and running as soon as he/she wakes up in the morning	☐	☐	☐	☐	☐
3. Your child prefers quiet, inactive games to more active ones	☐	☐	☐	☐	☐
4. Your child cries easily	☐	☐	☐	☐	☐
5. Your child gets upset (or sad) easily	☐	☐	☐	☐	☐
6. Your child reacts intensely when upset	☐	☐	☐	☐	☐
7. Your child is very sociable	☐	☐	☐	☐	☐
8. Your child takes a long time to warm up to strangers	☐	☐	☐	☐	☐
9. Your child is very friendly with strangers	☐	☐	☐	☐	☐
10. Your child prefer playing with others rather than alone	☐	☐	☐	☐	☐
11. Your child likes to be with people	☐	☐	☐	☐	☐
12. Your child finds other people more stimulating than anything else	☐	☐	☐	☐	☐

38. About the child's abilities and skills compared to peers.

Enter a cross from 1 - 5 for each line according to how well the statement fits your child.

	Very much lower 1	2	Typical for age 3	4	Very much higher 5
1. My child's ability to ask questions properly is:	☐	☐	☐	☐	☐
2. My child's ability to answer questions properly is:	☐	☐	☐	☐	☐
3. My child's ability to say sentences clearly enough to be understood by strangers is:	☐	☐	☐	☐	☐
4. The number of words my child knows is:	☐	☐	☐	☐	☐
5. My child's ability to use his/her words correctly is:	☐	☐	☐	☐	☐
6. My child's ability to get his/her message across to others when talking is:	☐	☐	☐	☐	☐
7. My child's ability to use the proper words when talking to others is:	☐	☐	☐	☐	☐
8. My child's ability to get what he/she wants by talking is:	☐	☐	☐	☐	☐
9. My child's ability to start a conversation, or start talking with other children is:	☐	☐	☐	☐	☐
10. My child's ability to keep a conversation going with other children is:	☐	☐	☐	☐	☐
11. The length of this child's sentences is:	☐	☐	☐	☐	☐
12. My child's ability to make "grown up" sentences is:	☐	☐	☐	☐	☐
13. My child's ability to correctly say the sounds in individual words is:	☐	☐	☐	☐	☐

39. About the child's behavior

The following list contains statements describing children's behavior and manners. To what extent are the following statements true of your child's behavior for the last 2 months

	Often/ typical	Sometimes	Never/ rarely
1. Afraid to try new things	☐	☐	☐
2. Can't concentrate, can't pay attention for long	☐	☐	☐
3. Can't sit still, restless or hyperactive	☐	☐	☐
4. Can't stand waiting; wants everything now	☐	☐	☐
5. Clings to adults or too dependent	☐	☐	☐
6. Cries a lot	☐	☐	☐
7. Defiant	☐	☐	☐
8. Demands must be met immediately	☐	☐	☐
9. Disturbed by any change in routine	☐	☐	☐
10. Doesn't eat well	☐	☐	☐
11. Doesn't seem to feel guilty after misbehaving	☐	☐	☐
12. Fears certain animals, situations or places	☐	☐	☐
13. Gets in many fights	☐	☐	☐
14. Gets into everything	☐	☐	☐
15. Gets too upset when separated from parents	☐	☐	☐
16. Hits others	☐	☐	☐
17. Nervous, highstrung, or tense	☐	☐	☐
18. Punishment doesn't change his/her behavior	☐	☐	☐
19. Quickly shifts from one activity to another	☐	☐	☐
20. Stomach aches or cramps (without medical cause)	☐	☐	☐
21. Too fearful or anxious	☐	☐	☐
22. Unhappy, sad or depressed	☐	☐	☐
23. Vomiting/ throwing up (without medical cause)	☐	☐	☐
24. Poorly coordinated or clumsy	☐	☐	☐
25. The child is teased/bullied by others	☐	☐	☐
26. Feelings are easily hurt	☐	☐	☐
27. Self-conscious or easily embarrassed	☐	☐	☐

40. How often does your child wake up during the night?

- ☐ 3 or more times per night
- ☐ 1-2 times per night
- ☐ A few times per week
- ☐ Seldom, never

41. Approximately, how many hours does the child usually sleep per night on weekdays?

- ☐ 8 hours or less
- ☐ 9 hours
- ☐ 10 hours
- ☐ 11 hours
- ☐ 12 hours or more

42. About your concerns

	No	Yes
1. Do you have any concerns about how your child speak and pronounce sounds?	☐	☐
2. Are you concerned because your child is demanding and difficult to cope with?	☐	☐
3. Are you concerned because your child is hardly interested at all in playing with other children?	☐	☐
4. Do you have any concerns because your child's activity level is so high?	☐	☐
5. Have others (family, nursery, health visitor) expressed concerns about your child's development?	☐	☐
6. Are you concerned because your child is hardly interested at all in playing with other children?	☐	☐

**If Yes:**	No	Yes a bit	Yes a lot
1. Is the child bothered or disturbed by the difficulties?	☐	☐	☐
2. Do the difficulties affect the child's daily life in any of the following areas:			
- At home/in the family	☐	☐	☐
- With friends/ peers	☐	☐	☐
- In the kindergarten/ outdoor nursery/ with child minder	☐	☐	☐
3. Do the difficulties cause strain on you or the family as a whole?	☐	☐	☐

4. If the child has difficulties, how old was the child when the difficulties started? ☐☐,

Questions about yourself

43. What is your current weight?

Weight ☐☐☐,☐ kg

44. Are you pregnant now?

☐ No ☐ Yes

45. What are the smoking habits in your houshold?

	You	Your partner/ spouse
1. Do not smoke	☐	☐
2. Smoke sometimes	☐	☐
3. Smoke daily	☐	☐
4. If daily - no. Of sigarettes per day	☐☐	☐☐

46. How often do you consume alcohol at present?

☐ Roughly 6-7 times a week
☐ Roughly 4-5 times a week
☐ Roughly 2-3 times a week
☐ Roughly once a week
☐ Roughly 1-3 times a month
☐ Less than once a month
☐ Never

47. How many alcohol units do you usually drink when you consume alcohol? Enter a cross for both weekends and weekdays. *(See explanation below about alcohol units.)*

	Weekends	Weekdays
10 or more	☐	☐
7-9	☐	☐
5-6	☐	☐
3-4	☐	☐
1-2	☐	☐
Less than 1	☐	☐

Alcohol units:
In order to compare different types of alcohol, we ask for the number of alcohol units (1,5 cl of pure alcohol). This means the following practice:

1 glass (1/3 litre) of beer	=	1 unit
1 wine glass of red or white wine	=	1 unit
1 wine glass of sherry or other fortified wine	=	1 unit
1 brandy glass of spirits of liqueur	=	1 unit
1 bottle of alcopop/cider	=	1 unit

48. Have you had a serious illness or health problems which has arisen during the last 5 years (Heart disease, cancer, muscle disease, serious chronic disease such as diabetes, mental illness, disability or other illness)?

☐ No ☐ Yes If No, go to question 50

If Yes,
Report which illness(es) and cross off whether a diagnosis has been given by a medical doctor and if you have been hospitalized for this illness.

Write the name of the illness/disorder	Doctor given a diagnosis: No	Doctor given a diagnosis: Yes	Hospitalization: No	Hospitalization: Yes	If you are well, about how old were you?
1. ____________	☐	☐	☐	☐	☐☐ year
2. ____________	☐	☐	☐	☐	☐☐ year
3. ____________	☐	☐	☐	☐	☐☐ year
4. ____________	☐	☐	☐	☐	☐☐ year

49. Has this or these illnesses/ problems made it difficult for you to function in your daily life, the last 5 years?

No	Yes a little	Yes a great deal	Yes very much
☐	☐	☐	☐

50. Have you ever had problems with your physical or mental health which has limitated in your work or social activities with friends or family?

☐ No ☐ Yes

If yes, how much have the problems affected you?

	Very much	A great deal	Some	A little	Not at all
1. Physical health	☐	☐	☐	☐	☐
2. Mental health	☐	☐	☐	☐	☐

51. Have you been bothered during the last 2 weeks by any of the following? *(Enter a cross for each line.)*

	Not bothered	A little bothered	Quite bothered	Very much bothered
1. Feeling fearful	☐	☐	☐	☐
2. Nervousness or shakiness inside	☐	☐	☐	☐
3. Feeling hopeless about the future	☐	☐	☐	☐
4. Feeling blue	☐	☐	☐	☐
5. Worrying too much about things	☐	☐	☐	☐
6. Feeling everything is an effort	☐	☐	☐	☐
7. Feeling tense or keyed up	☐	☐	☐	☐
8. Suddenly scared for no reason	☐	☐	☐	☐

52. If you have a husband/ boyfriend/ partner, How much do you agree with these descriptions of your relationship with your husband/ partner? *(Enter a cross for each line.)*

	Completely agree	Agree	Agree somewhat	Disagree somewhat	Disagree	Totally disagree
1. My partner and I have problems in our relationship	☐	☐	☐	☐	☐	☐
2. I am very happy in my relationship	☐	☐	☐	☐	☐	☐
3. My partner is generally understanding	☐	☐	☐	☐	☐	☐
4. I am satisfied with the relationship with my partner	☐	☐	☐	☐	☐	☐
5. We agree on how children should be raised	☐	☐	☐	☐	☐	☐

53. How often does this happen in your home? *(Enter a cross for each line)*

	Never	Almost never	Some-times	Often	Always
1. You let your child know when he/she is doing a good job with something	☐	☐	☐	☐	☐
2. You threaten to punish your child and then do not actually punish him/her	☐	☐	☐	☐	☐
3. You have a friendly talk with your child	☐	☐	☐	☐	☐
4. Your child talkes him/herself out of being punished after he/she has done something wrong	☐	☐	☐	☐	☐
5. You ask you child about his/her day in childcare	☐	☐	☐	☐	☐
6. You compliment your child when he/she does something well	☐	☐	☐	☐	☐
7. You praise your child if he/she behaves well	☐	☐	☐	☐	☐
8. You talk to your child about his/her friends	☐	☐	☐	☐	☐
9. You let your child out of a punishment early (E.g. Lift restrictions earlier than you originally said)	☐	☐	☐	☐	☐

54. Make a cross whether you agree or disagree with the following statements
(Enter a cross for each line from totally disagree to totally agree.)

	Totally disagree	Disagree	Slightly disagree	Neither agree nor disagree	Slightly agree	Agree	Totally agree
1. In most ways my life is close to my ideal	☐	☐	☐	☐	☐	☐	☐
2. The conditions of my life are excellent	☐	☐	☐	☐	☐	☐	☐
3. I'm satisfied with my life	☐	☐	☐	☐	☐	☐	☐
4. So far I have gotten the important things I want in life	☐	☐	☐	☐	☐	☐	☐
5. If I could live my life over, I would change almost nothing	☐	☐	☐	☐	☐	☐	☐
6. I really enjoy my work	☐	☐	☐	☐	☐	☐	☐

55. Have you, during the last year, experienced any of the following situations?

	No	Yes, during the last year	Yes, 2-5 years ago
1. Have you had problems at work or where you study?	☐	☐	☐
2. Have you had financial problems?	☐	☐	☐
3. Have you been divorced, separated or ended your relationship with your partner?	☐	☐	☐
4. Have you had problems or conflicts with family, friends or neighbors?	☐	☐	☐
5. Have you been seriously worried that there is something wrong with the child?	☐	☐	☐
6. Have you been seriously ill or injured?	☐	☐	☐
7. HHas anyone close to you been seriously ill or injured?	☐	☐	☐
8. Have you been involved in a serious accident, fire or robbery?	☐	☐	☐
9. Have you been the victim of maltreatment or abuse?	☐	☐	☐
10. Have you lost someone close to you?	☐	☐	☐
11. Other dramatic events/experiences you have had:	☐	☐	☐

Describe: ______________________________

56. Has any of the events listed in the questions aboveaffected you so that you have been on sick leave or not been able to function in your daily life/ work?

☐ No ☐ Yes

The list below consists of many statements that may fit or not fit as a description of you/your person. Cross off on each line for how you think each statement fit as a description of yourself. If you think a question is difficult to answer, you can skip it and continue with the next question.

57. Describe yourself the way you usually are: *(Enter a cross for each line)*

	Strongly disagree	disagree somewhat	Neither nor	Agree somewhat	Strongly agree
1. Liven up in a party	☐	☐	☐	☐	☐
2. Care little about others	☐	☐	☐	☐	☐
3. Am always well prepared	☐	☐	☐	☐	☐
4. Become easlily distressed	☐	☐	☐	☐	☐
5. Have a rich vocabulary	☐	☐	☐	☐	☐
6. Do not say much	☐	☐	☐	☐	☐
7. Am interested in other people	☐	☐	☐	☐	☐
8. Leave things lying around	☐	☐	☐	☐	☐
9. Am usually relaxed	☐	☐	☐	☐	☐
10. Have problems understanding abstract ideas	☐	☐	☐	☐	☐
11. Feel at ease with other people	☐	☐	☐	☐	☐
12. Offend people	☐	☐	☐	☐	☐
13. Am attentive to detail	☐	☐	☐	☐	☐
14. Worry about many things	☐	☐	☐	☐	☐
15. Have a lively imagination	☐	☐	☐	☐	☐
16. Stay in the background	☐	☐	☐	☐	☐
17. Have empathy with other people	☐	☐	☐	☐	☐
18. Mess things up	☐	☐	☐	☐	☐
19. Rarely feel in low spirits	☐	☐	☐	☐	☐
20. Am not interested in abstract ideas	☐	☐	☐	☐	☐
21. Initiate conversations	☐	☐	☐	☐	☐
22. Am not interested in other peoples' problems	☐	☐	☐	☐	☐
23. Complete tasks at once	☐	☐	☐	☐	☐
24. Am easily interrupted	☐	☐	☐	☐	☐
25. Have excellent ideas	☐	☐	☐	☐	☐
26. Have little to say	☐	☐	☐	☐	☐
27. Am good-natured	☐	☐	☐	☐	☐
28. Often forget to put things back	☐	☐	☐	☐	☐
29. Become easily upset	☐	☐	☐	☐	☐
30. Har ikke god forestillingsevne	☐	☐	☐	☐	☐
31. Do not have a good imagination	☐	☐	☐	☐	☐
32. Am not interested in other people	☐	☐	☐	☐	☐
33. Like order and tidiness	☐	☐	☐	☐	☐
34. Lot of mood changes	☐	☐	☐	☐	☐
35. Am quick to understand things	☐	☐	☐	☐	☐
36. Do not like to attract attention	☐	☐	☐	☐	☐
37. Take time to help others	☐	☐	☐	☐	☐
38. Shirk from responsibilities	☐	☐	☐	☐	☐
39. Often have mood swings	☐	☐	☐	☐	☐
40. Often use difficult words	☐	☐	☐	☐	☐
41. Have nothing against being the centre of attention	☐	☐	☐	☐	☐
42. Am sensitive to other peoples' feelings	☐	☐	☐	☐	☐
43. Perform according to plan	☐	☐	☐	☐	☐
44. Become easily irritated	☐	☐	☐	☐	☐
45. Use time to think things over	☐	☐	☐	☐	☐

	Strongly disagree	disagree somewhat	Neither nor	Agree somewhat	Strongly agree
46. Am quiet in company with strangers	☐	☐	☐	☐	☐
47. Put others at their ease	☐	☐	☐	☐	☐
48. Am thorough in my work	☐	☐	☐	☐	☐
49. Often feel down	☐	☐	☐	☐	☐
50. Am full of ideas	☐	☐	☐	☐	☐

58. We wish to prepare for child care research in MoBa, and want to look at the connections between child care quality and health. We therefore ask you to name the child's present or previous kindergarten, when the child went there, and in what municipality the kindergarten is placed. This will enable us to gather information from a public kindergarten register (BASIL) so that we can compare different kindergartens based on number of employees, number of employees with Early Childhood Education, kindergarten size, and other resources.

My child has never attended kindergarten ☐

Start with the first kindergarten the child attended

Name of the kindergarten	Municipality			
(F.eks Kløverenga barnehage)	*(Nes)*	*(Fall X*	*Spring ☐*	*Year 2 0 0 9)*
1.	....	Fall ☐	Spring ☐	Year
2.	....	Fall ☐	Spring ☐	Year
3.	....	Fall ☐	Spring ☐	Year
4.	....	Fall ☐	Spring ☐	Year

Comments

Have you remembered to fill in the date on which you completed the questionnaire on page 1?

Thank you very much for your continued participation in The Norwegian Mother and Child Cohort Study.
