# Supplementary material for: Association between colic and sleep problems in infancy and subsequent development, emotional and behavioral problems: a longitudinal study
Source: BMC Pediatr. 2021 Jan 7;21:23. doi: 10.1186/s12887-020-02483-1 (PMC7788887; doi:10.1186/s12887-020-02483-1)
Supplement: Supplementary file 3 — Additional file 3. [file 12887_2020_2483_MOESM3_ESM.pdf]

den norske **Mor & barn undersøkelsen**

Questionnaire 6 – Your child at 36 months

In this questionnaire we will ask you some questions which you may recognise from previous questionnaires. We do this because we want to continue following your and your child's development. You are welcome to consult your child's Health card so that you can use the information contained in it.

If you feel that a question is too upsetting or difficult to answer you can skip this question and go on to the next one.

The questionnaire will be processed by a computer. It is therefore important that you follow these instructions when completing it:

- Use a blue or black ballpoint pen.
- Put a *cross* in the box that is most relevant like this: ☒
- If you put a cross in the wrong box, correct it by filling in the box completely like this: ■
- Write *numbers* in the large boxes. ***It is important that you only write in the white area of each box like this:***

  Number: 1 2 3 4 5 6 7 8 9 0

- Numbered boxes have two or more squares. When you enter a single-digit number, use the square on the right.

  Example: 5 is entered as follows 5

- Specific information concerning, *for example, medication* should be written on the lines provided. *Write clearly in CAPITAL LETTERS.*
- Remember to fill in the date on which you completed the questionnaire

As soon as you have completed this questionnaire, return it to us in the stamped addressed envelope provided.

Specify the day, month and year when the questionnaire was completed — Day / Month / Year *(write the year in full, e.g. 2005)*

Your child's development, health and history of illness

1. What is your child's height and weight (without clothes) at 3 years? If you know your child's height and weight at 2 years and 15-18 months, enter these measurements too. (If you don't know them, go on to the next question.) Give the date when the measurements were taken and enter a cross to indicate whether they were taken by you.

	Date of measurement (Day / Month / Year)	Height	Weight	Own measurement
Approx. 3 years		. cm	. kg	☐
Approx. 2 years		. cm	. kg	☐
Approx. 15-18 months		. cm	. kg	☐

2. How many months old was your child when he/she took his/her first steps unaided? mth — Still not walking unaided. ☐

Questionnaire 6C - MB - 25.000 - 0407 - Bording

The following questions concern any illnesses or health problems your child has had. We will first ask you about longer-term problems and then about illnesses and problems of a more acute nature.

3. Has your child suffered any long-term illness or health problems since the age of 18 months?

Health problem	No	Yes, has now	Yes, had previously	If so, has child been referred to a specialist: No	Yes
1. Impaired hearing	☐	☐	☐	☐	☐
2. Impaired vision	☐	☐	☐	☐	☐
3. Delayed motor development (e.g. sits/walks late)	☐	☐	☐	☐	☐
4. Cerebral palsy	☐	☐	☐	☐	☐
5. Joint problems	☐	☐	☐	☐	☐
6. Diabetes	☐	☐	☐	☐	☐
7. Gained too little weight	☐	☐	☐	☐	☐
8. Gained too much weight	☐	☐	☐	☐	☐
9. Heart defect	☐	☐	☐	☐	☐
10. Testicles not descended into scrotum	☐	☐	☐	☐	☐
11. Asthma	☐	☐	☐	☐	☐
12. Allergy affecting eyes or nose, e.g. hay fever	☐	☐	☐	☐	☐
13. Atopic eczema (childhood eczema)	☐	☐	☐	☐	☐
14. Other type of eczema	☐	☐	☐	☐	☐
15. Frequent diarrhoea	☐	☐	☐	☐	☐
16. Frequent stomach pains	☐	☐	☐	☐	☐
17. Food allergy/intolerance	☐	☐	☐	☐	☐
18. Other gastrointestinal problems	☐	☐	☐	☐	☐
19. Late or abnormal speech development	☐	☐	☐	☐	☐
20. Sleep problems	☐	☐	☐	☐	☐
21. Trouble relating to others	☐	☐	☐	☐	☐
22. Hyperactivity	☐	☐	☐	☐	☐
23. Autistic traits	☐	☐	☐	☐	☐
24. Other behavioural problems	☐	☐	☐	☐	☐
25. Other long-term illness/condition	☐	☐	☐	☐	☐

Specify ______________________________

4. If your child has been to see a specialist or to the hospital, what did the investigation show?

☐ Everything was fine
☐ Still some doubts/further investigations needed
☐ Has not been for any investigation yet

☐ Received diagnosis I: ______________________________

____

☐ Received diagnosis II: ______________________________

____

☐ Received diagnosis III: ______________________________

____

5. If your child has a serious or long-term illness, describe it, if possible, in more detail:

____

____

6. Has your child ever been exposed to or involved in a serious incident?

☐ No ☐ Yes

7. If yes, give a description:

____

____

____

____

____

____

8. Do you think that this has affected your child's behaviour or development?

☐ No ☐ Yes

9. Has your child suffered any acute illness/health problem since the age of 18 months?
(Specify how many times and whether your child has been admitted to or examined at a hospital for this health problem.)

	No	Yes	Number of times	If yes, has child been admitted to or examined in hospital? No	Yes
1. Common cold	☐	☐	☐☐	☐	☐
2. Throat infection with a confirmed streptococci	☐	☐	☐☐	☐	☐
3. Other type of throat infection	☐	☐	☐☐	☐	☐
4. Ear infection	☐	☐	☐☐	☐	☐
5. Pseudocroup	☐	☐	☐☐	☐	☐
6. Bronchitis	☐	☐	☐☐	☐	☐
7. Pneumonia	☐	☐	☐☐	☐	☐
8. Gastric flu/diarrhoea	☐	☐	☐☐	☐	☐
9. Urinary tract infection	☐	☐	☐☐	☐	☐
10. Encephalitis/meningitis	☐	☐	☐☐	☐	☐
11. Febrile convulsions	☐	☐	☐☐	☐	☐
12. Other convulsions (without any fever)	☐	☐	☐☐	☐	☐
13. Injury or accident	☐	☐	☐☐	☐	☐
14. Other ________________	☐	☐	☐☐	☐	☐

10. If your child has been examined in or admitted to hospital, give the name of the hospital:

Hospital name: ______________________

Hospital name: ______________________

Hospital name: ______________________

11. Has your child been referred to the following services since the age of 18 months?

	No	Yes
Habilitation service	☐	☐
Educational psychology service	☐	☐
Child psychiatric clinic/department	☐	☐

12. Has your child taken any medication during the last 12 months? *(This means any type of medication, including fever-reducing medicines, alternative medicines and herbal remedies)*

☐ No ☐ Yes

13. If yes, give the name of the medicines and indicate how long your child took these medicines for altogether and whether he/she is still taking them now.

Name of medicine: (CAPITALS)	Duration of use					Still being taken now?	
	0-2 weeks	3-4 weeks	1-2 mth	3-6 mth	7-12 mth	No	Yes
____________________	☐	☐	☐	☐	☐	☐	☐
____________________	☐	☐	☐	☐	☐	☐	☐
____________________	☐	☐	☐	☐	☐	☐	☐
____________________	☐	☐	☐	☐	☐	☐	☐

14. Has your child been given any vaccinations since you completed the previous questionnaire (at around 18 months or 6 months)? ☐ No ☐ Yes

15. If yes, specify which vaccinations and when your child received them.

Type of vaccination:	Date given: Day	Month	Year
____________________	☐☐	☐☐	☐☐
____________________	☐☐	☐☐	☐☐

16. Is your child taking at the moment any cod liver oil, vitamins or other dietary supplements?

	Yes, daily	Sometimes	No
1. Cod liver oil	☐	☐	☐
2. Fluoride tablets	☐	☐	☐
3. Vitamin preparations, specify ____________	☐	☐	☐
4. Iron supplement, specify ____________	☐	☐	☐
5. Other dietary supplements, specify____________	☐	☐	☐

Your child's development and ability to cope

In this section you will find some questions repeated in a different form. We do this so that we can compare your child's development with other similar studies and try out the best way to ask the question. The questions will relate to children who have reached different stages of development. Answer all the questions as well as you can, even if everything does not necessarily apply to your child.

✓ ASQ

17. About your child's motor development. *(Enter a cross in a box for each item.)*

	Yes	A few times	Not yet
1. Can your child kick a ball by swinging his/her leg forward without holding onto anything for support?	☐	☐	☐
2. Can your child catch a large ball with both hands?	☐	☐	☐
3. When drawing, does your child hold a pencil, crayon or pen between his/her fingers and thumb like an adult does?	☐	☐	☐
4. Can your child undo one or more buttons?	☐	☐	☐

18. About your child's language skills. *(Enter a cross for the option which best describes the way your child talks.)*

- ☐ Not yet talking
- ☐ He/she is talking, but you can't understand him/her
- ☐ Talking in one-word utterances, such as "milk" or "down"
- ☐ Talking in 2- to 3-word phrases, such as "me got ball" or "give doll"
- ☐ Talking in fairly complete sentences, such as "I got a doll" or "can I go outside?"
- ☐ Talking in long and complicated sentences, such as "when I went to the park, I went on the swings" or "I saw a man standing on the corner"

19. Your child's body language. *(Enter a cross in the box of the answer that fits your child best for each statement.)*

	Yes, usually	Very seldom	Not yet
1. When you enthusiastically say: "Where is the ball (or other toy)?", will your child point towards the toy, even if it is more than 1 metre away?	☐	☐	☐
2. When you look at a distant object and, surprised and excited, say: "Waoo...what's that?", - does he/she turn his/her head in the same direction as you?	☐	☐	☐
3. Does your child use sounds or words together with gestures? (for example, uses sounds when pointing or reaching towards toys or objects)	☐	☐	☐
4. Does your child show you toys by looking at you and holding the toy up towards you? (from a distance just so you can look at it)	☐	☐	☐

20. About your child's social skills.
(Enter a cross in a box for each statement to indicate whether you agree or disagree.)

	Disagree	Partially agree	Totally agree
1. Your child shares readily with other children (treats, toys, pencils, etc.)	☐	☐	☐
2. Your child is helpful if someone is hurt, upset or feeling ill	☐	☐	☐
3. Your child is considerate of other people's feelings	☐	☐	☐
4. Your child is kind to younger children	☐	☐	☐
5. Your child often volunteers to help others (parents, teachers, other children)	☐	☐	☐
6. Your child pays careful attention when you try to teach him/her something new	☐	☐	☐

✓ ASQ

21. Understanding what others say and being able to communicate
(Enter a cross in the box of the answer that fits your child best for each statement.)

	Yes	A few times	Not yet
1. Without showing him/her first, does your child point to the correct picture when you say, "Where is the cat" or "Where is the dog"? Your child must only point at the correct picture.	☐	☐	☐
2. When you ask your child to point at his/her eyes, nose, hair, feet, ears, etc., does he/she point correctly at least seven parts of the body? (The child can point at himself/herself, you or a doll.)	☐	☐	☐
3. Does your child use sentences made up of three or four words?	☐	☐	☐
4. Without giving him/her help by pointing or using gestures, ask your child to "Put the shoe on the table" and "Put the book under the chair". Does your child carry out both of these directions correctly? .	☐	☐	☐
5. When looking at a picture book, does your child tell you what is happening or what action is taking place in the picture? (For example, "Barking", "Running", "Eating" and "Crying"?) You may ask, "What is the dog (or boy) doing?"	☐	☐	☐
6. Can your child tell you at least two things about an object he/she is familiar with? If you say, for example, "Tell me about your ball", will your child answer by saying something like "It is round, I can throw it, it is big"?	☐	☐	☐

22. About body language and other ways of communicating with others. *(We are asking you about how your child usually is. If the behaviour is rare , e.g. you have only seen it once or twice, enter a cross in the 'No' box. Enter a cross in a box for each question.)*

	Yes	No
1. Does your child respond to his/her name one of the first two times you call?	☐	☐
2. Does your child ever bring objects over to you to show you something?	☐	☐
3. Does your child imitate you (e.g. you make a face - will your child imitate it?)?	☐	☐
4. Does your child ever use his/her index finger to point, to indicate interest in something?	☐	☐
5. Does your child take an interest in other children?	☐	☐
6. If you point at a toy across the room, does your child look at it?	☐	☐
7. Is it easy to make eye contact with your child?	☐	☐
8. Does your child react when spoken to, for instance, by looking, listening, smiling, speaking or babbling?	☐	☐
9. Does you child ever seem oversensitive to noise (e.g. plugging ears)?	☐	☐
10. Does you child only choose a very small number of particular toys or objects, even if you try to make him/her interested in more things?	☐	☐
11. Does your child wave to people to greet or say goodbye to them?	☐	☐
12. Can your child hurt himself/herself a lot without seeming to be bothered (has a high pain threshold)?	☐	☐

23. About talking with others. *(Enter a cross in a box for each question to indicate whether you think it applies to your child or not.)*

	Yes	No
1. Does your child talk using short phrases or sentences?	☐	☐
2. Do you have a to-and-fro "conversation" with your child that involves taking turns or building on what you have said?	☐	☐
3. Does your child ever use odd phrases or say the same thing over and over again in almost exactly the same way? (either phrases that the child hears other people use or ones that he/she makes up)	☐	☐
4. Does your child ever use socially inappropriate questions or statements? For example, does your child ever regularly ask personal questions or make personal comments at awkward times?	☐	☐
5. Does your child ever get his/her pronouns mixed up (i.e. saying "you" or "he/she" instead of "I")?	☐	☐
6. Does your child ever use words that he/she seems to have invented or made up himself/herself, put things in odd, indirect ways or use metaphorical ways of saying things? (e.g. saying "hot rain" for "steam")	☐	☐
7. Does your child ever say the same thing over and over in exactly the same way or insist that you say the same thing over and over again?	☐	☐
8. Does your child ever have things that he/she seems to have to do in a very particular way or order, or rituals that the child insists that you go through?	☐	☐

24. About behaviour and specific things that children can think of doing. *(Enter a cross in a box for each question to indicate whether you think it applies to your child or not.)*

	Yes	No
9. Does your child's facial expression usually seem appropriate to the particular situation, as far as you can tell?	☐	☐
10. Does your child ever use your hand like a tool or as if it were part of his/her own body (e.g. pointing with your finger or putting your hand on a doorknob to get you to open the door)?	☐	☐
11. Does your child ever have any interests that preoccupy him/her and might seem odd to other people (e.g. traffic lights, drainpipes or timetables)?	☐	☐
12. Does your child ever seem to be more interested in parts of a toy or an object, rather than in using the object as it was intended (e.g. spinning the wheels of a car)?	☐	☐
13. Does your child ever have any special interests that are unusual in their intensity, but otherwise appropriate for his/her age and peer group (e.g. trains or dinosaurs)?	☐	☐
14. Does your child ever seem to be unusually interested in the sight, feel, sound, taste or smell of things or people?	☐	☐
15. Does your child ever have any mannerisms or odd ways of moving his/her hands or fingers, such as flapping or moving his/her fingers in front of his/her eyes?	☐	☐
16. Does your child ever have any complicated movements of his/her whole body, such as spinning or repeatedly bouncing up and down?	☐	☐
17. Does your child ever injure himself/herself deliberately, such as by biting his/her arm or banging his/her head?	☐	☐
18. Does your child ever have any objects that he/she has to carry around (other than a soft toy or comfort blanket)?	☐	☐

25. About your child's social development and interest in others. *(Enter a cross in a box for each question to indicate whether you think it applies to your child or not.)*

	Yes	No
19. Does your child have any particular friends or a best friend?	☐	☐
20. Does your child ever talk with you just to be friendly (rather than to get something)?	☐	☐
21. Does your child ever spontaneously copy you (or other people) or what your are doing (such as vacuuming, gardening or mending things)?	☐	☐
22. Does your child ever spontaneously point at things around him/her just to show you things (not because he/she wants them)?	☐	☐
23. Does your child ever use gestures, other than pointing or pulling your hand, to let you know what he/she wants?	☐	☐
24. Does your child nod his/her head to indicate yes?	☐	☐
25. Does your child shake his/her head to indicate no?	☐	☐
26. Does your child usually look at you directly in the face when doing things with you or talking with you?	☐	☐
27. Does your child smile back if someone smiles at him/her?	☐	☐
28. Does your child ever show you things that interest him/her to engage your attention?	☐	☐

cont. next page

Material for SCQ is protected by copyright © 2005 by Western Psychological Services. Registered translation printed by the Norwegian Institute of Public Health; permission is given only for its use in the ABC Project by the publisher, Western Psychological Services, 12031 Wilshire Boulevard, Los Angeles, California 90025-1251, U.S.A. All rights are binding under international law. No further reprinting is permitted, either in whole or in part, without having obtained prior authorisation from Western Psychological Services (weinberg@wpspublish.com)

	Yes	No
29. Does your child ever offer to share things other than food with you?	☐	☐
30. Does your child ever seem to want you to join in his/her enjoyment of something?	☐	☐
31. Does your child ever try to comfort you when you are sad or hurt?	☐	☐
32. If your child wants something or wants help, does he/she look at you and use gestures with sounds or words to get your attention?	☐	☐
33. Does your child show a normal range of facial expressions?	☐	☐
34. Does your child ever spontaneously join in and try to copy the actions in social games, such as “The Mulberry Bush” or “London Bridge is Falling Down”?	☐	☐
35. Does your child play any pretend or make-believe games?	☐	☐
36. Does your child seem interested in other children of approximately the same age whom he/she does not know?	☐	☐
37. Does your child respond positively when another child approaches him/her?	☐	☐
38. If you come into a room and start talking to your child without calling his/her name, does he/she usually look up and pay attention to you?	☐	☐
39. Does your child ever play imaginative games with another child in such a way that you can tell that each child understands what the other is pretending?	☐	☐
40. Does your child play cooperatively in games that need some form of joining in with a group of other children, such as hide-and-seek or ball games?	☐	☐

26. Loss of skills. *(Is there something your child used to be able to do, but has lost the ability to do?)*

	No	Yes	Not sure
1. Has your child lost any language skills? (For example, used single words or sentences for a time and then stopped using the words)	☐	☐	☐
2. Has your child lost any social skills? (For example, could wave or say “Hi” to greet someone, then lost this skill)	☐	☐	☐
3. Has your child turned out to be less sociable? (For example, he/she is more difficult to have eye contact with, is less interested in other people now)	☐	☐	☐
4. Has your child lost any motor skills? (For example, could run and jump while remaining steady, but falls over much more now)	☐	☐	☐

Your child’s temperament and behaviour

27. To what extent do the following statements apply to your child’s behaviour during the last two months? *(Enter a cross in a box for each item.)*

	Very typical	Quite typical	Neither/ nor	Not so typical	Not at all typical
1. Your child cries easily	☐	☐	☐	☐	☐
2. Your child is always on the go	☐	☐	☐	☐	☐
3. Your child prefers playing with others rather than alone	☐	☐	☐	☐	☐
4. Your child is off and running as soon as he/she wakes up in the morning	☐	☐	☐	☐	☐
5. Your child is very sociable	☐	☐	☐	☐	☐
6. Your child takes a long time to warm up to strangers	☐	☐	☐	☐	☐
7. Your child gets upset or sad easily	☐	☐	☐	☐	☐
8. Your child prefers quiet, inactive games to more active ones	☐	☐	☐	☐	☐
9. Your child likes to be with people	☐	☐	☐	☐	☐
10. Your child reacts intensely when upset	☐	☐	☐	☐	☐
11. Your child is very friendly with strangers	☐	☐	☐	☐	☐
12. Your child finds other people more fun than anything else	☐	☐	☐	☐	☐
13. Your child complains that certain garments are too tight	☐	☐	☐	☐	☐
14. Your child is distressed by having his/her face or hair washed	☐	☐	☐	☐	☐

✓ CBCL

28. The following list contains statements describing children's behaviour and manner from the age of 2-3. Some of these features are temporary while others continue for a longer period of time. To what extent are the following statements true of your child's behaviour during the last two months? *(Enter a cross in a box for each item.)*

	Not true	Somewhat or sometimes true	Very true or often true
1. Afraid to try new things	☐	☐	☐
2. Can't concentrate, can't pay attention for long	☐	☐	☐
3. Can't sit still, restless or hyperactive	☐	☐	☐
4. Can't stand waiting, wants everything now	☐	☐	☐
5. Clings to adults or too dependent	☐	☐	☐
6. Constipated, doesn't move bowels	☐	☐	☐
7. Defiant	☐	☐	☐
8. Demands must be met immediately	☐	☐	☐
9. Disturbed by any change in routine	☐	☐	☐
10. Doesn't want to sleep alone	☐	☐	☐
11. Doesn't eat well	☐	☐	☐
12. Doesn't seem to feel guilty after misbehaving	☐	☐	☐
13. Eats or drinks things that are not food (don't include sweets)	☐	☐	☐
14. Gets in many fights	☐	☐	☐
15. Gets into everything	☐	☐	☐
16. Gets too upset when separated from parents	☐	☐	☐
17. Hits others	☐	☐	☐
18. Poorly coordinated or clumsy	☐	☐	☐
19. Punishment doesn't change his/her behaviour	☐	☐	☐
20. Quickly shifts from one activity to another	☐	☐	☐
21. Resists going to bed at night	☐	☐	☐
22. Stomach aches or cramps (without medical cause)	☐	☐	☐
23. Sudden changes in moods or feelings	☐	☐	☐
24. Too fearful or anxious	☐	☐	☐
25. Vomiting, throwing up (without medical cause)	☐	☐	☐
26. Doesn't seem to be happy eating food (don't include sweets)	☐	☐	☐

29. Some more statements follow about your child's behaviour and manner. We are again asking to what extent you feel the statements are true of your child during the last two months? *(Enter a cross in a box for each item.)*

	Not true	Somewhat or sometimes true	Very true or often true
1. Becomes distracted or diverted by outside stimuli (sounds or events)	☐	☐	☐
2. Finds it difficult waiting his/her turn	☐	☐	☐
3. Has problems keeping focused on tasks or activities	☐	☐	☐
4. Is excessively talkative	☐	☐	☐
5. Doesn't differentiate between adults; behaves the same way to all of them	☐	☐	☐
6. Will wander after other adults, even if they are strangers	☐	☐	☐
7. Doesn't seem to listen when he/she is being spoken to	☐	☐	☐
8. Has a habit of rolling his/her head around or making humming sounds	☐	☐	☐
9. Mood can vary greatly from day to day	☐	☐	☐
10. Is extremely passive, needs help to get going	☐	☐	☐
11. "Tests" other children to see whether they get angry	☐	☐	☐
12. Becomes aggressive when he/she is frustrated	☐	☐	☐
13. His/her body is affected by twitches or contortions that seem difficult to control (e.g. eyes, mouth, nose or legs)	☐	☐	☐
14. Hits, shoves, kicks and bites other children (not including siblings)	☐	☐	☐
15. Is very anxious about getting dirty	☐	☐	☐
16. Wants things to be clean and tidy	☐	☐	☐
17. Places toys or other objects in a certain order/sequence over and over again	☐	☐	☐
18. Wakes up in the night and needs help to get back to sleep	☐	☐	☐
19. Gets distressed when you go out and he/she is going to be looked after by family or a babysitter he/she knows	☐	☐	☐

cont. next page

	Not true	Somewhat or sometimes true	Very trueor often true
20. Does things he/she is not allowed to do to attract attention from adults	☐	☐	☐
21. Seems to have less fun than other children	☐	☐	☐
22. Is extremely noisy. Shouts and screams a lot	☐	☐	☐
23. Is disobedient or defiant (e.g. refuses to do anything you ask)	☐	☐	☐
24. Comes over to you when something happens that makes him/her afraid or anxious	☐	☐	☐
25. Runs off when you are outside	☐	☐	☐
26. Seems to have less energy	☐	☐	☐
27. Is very fussy when it comes to food	☐	☐	☐
28. Seems to be unhappy, sad or depressed	☐	☐	☐
29. Wakes up several times during the night	☐	☐	☐

30. About your child's eating habits and appetite and your attitude to it.

	Totally disagree	Slightly disagree	Neither/ nor	Slightly agree	Totally agree
1. I have to be sure that my child does not eat too many sweet things (sweets, ice cream, cakes or pastries)	☐	☐	☐	☐	☐
2. I have to be sure that my child does not eat too many high-fat foods	☐	☐	☐	☐	☐
3. I have to be sure that my child does not eat too much of his/her favourite food	☐	☐	☐	☐	☐
4. I intentionally keep some foods out of my child's reach	☐	☐	☐	☐	☐
5. I offer sweet things (sweets, ice cream, cakes, pastries) to my child as a reward for good behaviour	☐	☐	☐	☐	☐
6. I offer my child his/her favourite foods in exchange for good behaviour	☐	☐	☐	☐	☐
7. If I did not guide or regulate my child's eating he/she would eat too many junk foods	☐	☐	☐	☐	☐
8. If I did not guide or regulate my child's eating he/she would eat too much of his/her favourite foods	☐	☐	☐	☐	☐
9. My child should always eat all of the food on his/her plate	☐	☐	☐	☐	☐
10. I have to be especially careful to make sure that my child eats enough	☐	☐	☐	☐	☐
11. If my child says: "I'm not hungry", I try to get him/her to eat anyway	☐	☐	☐	☐	☐
12. If I did not guide or regulate my child's eating, he/she would eat much less than he/she should.	☐	☐	☐	☐	☐

31. About your concerns.

	No	Yes
1. Are you concerned because your child is demanding and difficult to cope with?	☐	☐
2. Have you every wondered if your child's hearing is impaired?	☐	☐
3. Have others (family, nursery, health visitor) expressed concerns about your child's development?	☐	☐
4. Are your concerned because your child is hardly interested at all in playing with other children?	☐	☐
5. Do you have any other concern about your child's health?	☐	☐

If so, specify ____

Your child's everyday life and environment

32. Do you live with your child's father?

☐ No ☐ Yes

33. If no, how much time does your child spend with his/her mother and father respectively?

	Mother	Father
More than half the time	☐	☐
Roughly half the time	☐	☐
At least once a week	☐	☐
At least once a month	☐	☐
Less often than once a month	☐	☐
Never	☐	☐

34. How often does your child have his/her teeth brushed?

☐ Twice a day or more
☐ Once a day
☐ Sometimes
☐ Never

35. Does your child use fluoride toothpaste?

☐ No
☐ Sometimes
☐ Yes, usually

36. Is your child ever present in a room where someone smokes?

- ☐ Yes, every day — Number of hours a day:
- ☐ Yes, several times a week
- ☐ Yes, sometimes
- ☐ Don't know
- ☐ No

37. How often is your child outside at present?

- ☐ Seldom
- ☐ Frequently, but less than 1 hour a day on average
- ☐ 1-3 hours a day on average
- ☐ More than 3 hours a day

38. How many hours on average does your child sit in front of a TV/video every day?

- ☐ 4 hours or more
- ☐ 3 hours
- ☐ 1-2 hours
- ☐ Less than 1 hour
- ☐ Seldom/never

39. How is your child cared for during the day at the moment? *(You can enter a cross in more than one box.)*

- ☐ At home with his/her mother
- ☐ At home with his/her father
- ☐ At home with an unqualified childminder
- ☐ At a childminder's
- ☐ In a short term outdoor day nursery
- ☐ In a day nursery

40. How many hours a week is your child looked after during the day by someone other than his/her mother or father?

Diet

41. How often does your child drink or eat the following at present? *(Select the frequency which is most applicable on average.)* *(Enter a cross in a box for each item.)*

	Seldom/ less than once a week	1-3 times a week	4-6 times a week	Once in 24 hrs	Twice in 24 hrs	3 times in 24 hrs	4 or more times in 24 hrs
1. Whole milk, sweet/sour	☐	☐	☐	☐	☐	☐	☐
2. Low-fat, extra low-fat, skimmed milk, sweet/sour	☐	☐	☐	☐	☐	☐	☐
3. Yogurt, natural	☐	☐	☐	☐	☐	☐	☐
4. Yogurt / yogurt drink with fruit	☐	☐	☐	☐	☐	☐	☐
5. Yogurt with active Lactobacillus, all types							
6. Juice	☐	☐	☐	☐	☐	☐	☐
7. Cordial / nectar / squash / fizzy drinks, sweetened	☐	☐	☐	☐	☐	☐	☐
8. Cordial / squash / fizzy drinks, with artificial sweeteners	☐	☐	☐	☐	☐	☐	☐
9. Meat filling (liver paste, ham, etc.)	☐	☐	☐	☐	☐	☐	☐
10. Fish filling (mackerel, caviar, etc.)	☐	☐	☐	☐	☐	☐	☐
11. Brown cheese, brown cheese spread	☐	☐	☐	☐	☐	☐	☐
12. Other types of cheese	☐	☐	☐	☐	☐	☐	☐
13. Jam, honey, chocolate spread, other sweet spread	☐	☐	☐	☐	☐	☐	☐
14. Eggs, boiled, fried, scrambled	☐	☐	☐	☐	☐	☐	☐
15. Other filling________________________	☐	☐	☐	☐	☐	☐	☐
16. Fruit	☐	☐	☐	☐	☐	☐	☐
17. Raisins	☐	☐	☐	☐	☐	☐	☐
18. Ice cream	☐	☐	☐	☐	☐	☐	☐
19. Ice lolly	☐	☐	☐	☐	☐	☐	☐
20. Biscuits	☐	☐	☐	☐	☐	☐	☐
21. Buns, cakes, waffles	☐	☐	☐	☐	☐	☐	☐
22. Chocolate	☐	☐	☐	☐	☐	☐	☐
23. Sweets, jelly babies, etc.	☐	☐	☐	☐	☐	☐	☐
24. Crisps, potato snacks	☐	☐	☐	☐	☐	☐	☐

42. How many slices of bread/crispbread does your child eat every day?

How many of these include fibre-rich bread/ crispbread (e.g. rye bread, Fedons bread)

43. How often does your child eat the following at present? *(Select the frequency which is most applicable on average.)* *(Enter a cross in a box for each item.)*

	Once a mth or less often	2-3 times a month	Once a week	Twice a week	3 times a week	4 times a week	5 or more times a week
1. Meat, rissoles, sausages, etc.	☐	☐	☐	☐	☐	☐	☐
2. Oily fish (salmon, herring, etc.)	☐	☐	☐	☐	☐	☐	☐
3. White fish (cod, coley, etc.)	☐	☐	☐	☐	☐	☐	☐
4. Fish pudding, fish cakes, fish balls, etc.	☐	☐	☐	☐	☐	☐	☐
5. Soup	☐	☐	☐	☐	☐	☐	☐
6. Pancakes	☐	☐	☐	☐	☐	☐	☐
7. Potatoes	☐	☐	☐	☐	☐	☐	☐
8. Pasta, spaghetti, noodles	☐	☐	☐	☐	☐	☐	☐
9. Pizza	☐	☐	☐	☐	☐	☐	☐
10. Rice	☐	☐	☐	☐	☐	☐	☐
11. Cooked vegetables	☐	☐	☐	☐	☐	☐	☐
12. Raw vegetables, salad	☐	☐	☐	☐	☐	☐	☐

Questions about yourself

44. What is your civil status at present?

☐ Married
☐ Cohabiting
☐ Single
☐ Separated/divorced
☐ Widowed
☐ Other

45. Are you in paid employment at the moment?

☐ No *(go to question 49)*
☐ Yes Usual number of hours per week: ☐☐,☐

46. What type of working pattern do you have? *(You can enter a cross in more than one box.)*

☐ Permanent day work
☐ Shift work/rota system
☐ Permanent afternoon/evening work
☐ Non-permanent (relief cover, relief on-call, supply, etc.)
☐ Permanent night work

47. How many days altogether were you absent from work last year (excluding holidays and time off in lieu)?

☐☐☐ days

48. What was the reason for this? *(You can enter a cross in more than one box.)*

☐ Leave
☐ Own illness, specify______________________________
☐ Sick child
☐ Other

49. Do you often feel lonely?

☐ Almost never
☐ Seldom
☐ Sometimes
☐ Generally
☐ Almost always

50. Do you have anyone other than your spouse /boyfriend/partner whom you can seek advice from in a difficult situation?

☐ No
☐ Yes, 1 or 2 people
☐ Yes, more than 2 people

51. How often do you see or talk on the telephone to your family (apart from your household) or close friends?

☐ Once a month or less
☐ 2-8 times a month
☐ More than twice a week

52. Have you ever experienced the following, since you became pregnant with this child, for a consecutive period of two weeks or more...... *(Enter a cross in a box for each item.)*

	No	Yes, during pregnancy	Yes, during first year after birth	Yes, during the last 2 years
1. Felt depressed, sad, down?	☐	☐	☐	☐
2. Had problems with your appetite or eaten too much?	☐	☐	☐	☐
3. Been affected by lethargy or a lack of energy?	☐	☐	☐	☐
4. Really got down on yourself and felt worthless?	☐	☐	☐	☐
5. Had problems concentrating or found it difficult to make decisions?	☐	☐	☐	☐
6. Had at least 3 of the problems mentioned above at the same time?	☐	☐	☐	☐

53. Are you pregnant now?

☐ No ☐ Yes

54. Have you had any long-term illness or health problems which have occurred during the last 3 years?

Physical problem:

☐ No

☐ Yes, before, describe: ____________________

☐ Yes, now, describe: ____________________

Mental problem:

☐ No

☐ Yes, before, describe: ____________________

☐ Yes, now, describe: ____________________

55. Have you yourself been examined at the hospital during the last 3 years?

☐ No

☐ Yes, which hospital? ____________________

56. Do you have any of the following problems at present; if so, how often and how much at a time? *(Enter a cross in a box for each item.)*

	How often do you have problems?					How much at a time?		
**Problems:**	Never	1–4 times a month	1–6 times a week	Once a day	More than once a day	Drops	Small gushes	Large amounts
1. Incontinence when coughing, sneezing or laughing	☐	☐	☐	☐	☐	☐	☐	☐
2. Incontinence during physical activity (running/jumping)	☐	☐	☐	☐	☐	☐	☐	☐
3. Incontinence with a strong need to urinate . .	☐	☐	☐	☐	☐	☐	☐	☐
4. Problems retaining faeces	☐	☐	☐	☐	☐			
5. Problems with flatulence								

57. How physically active are you? We are asking you here about the duration of activities where you get out of breath or sweat. How often does this happen? Include activities both at home and at work. *(Enter a cross in a box for each item.)*

	How often					
**Duration of activity where you get out of breath or sweat**	Never	Less than once a week	Once a week	Twice a week	3-4 times a week	5 times or more a week
Less than 30 minutes	☐	☐	☐	☐	☐	☐
Between 30 and 60 minutes	☐	☐	☐	☐	☐	☐
More than 60 minutes	☐	☐	☐	☐	☐	☐

58. Overall, how would you describe your physical health?

☐ Very good
☐ Good
☐ Poor
☐ Very poor

59. Do you smoke at present?

☐ Don't smoke

☐ Smoke sometimes - no. cigarettes per week: ☐☐☐

☐ Smoke every day - no. cigarettes per day: ☐☐☐

60. Do you take:

☐ Chewing tobacco/snuff
☐ Nicotine chewing gum
☐ Nicotine patches
☐ Nicotine inhaler

61. How often do you consume alcohol at present?

☐ Roughly 6–7 times a week
☐ Roughly 4–5 times a week
☐ Roughly 2-3 times a week
☐ Roughly once a week
☐ Roughly 1-3 times a month
☐ Less than once a month
☐ Never

62. How many alcohol units do you usually drink when you consume alcohol? *(Enter a cross for both weekends and week-days)* (See explanation below about alcohol units.)

	Weekend	Weekdays
10 or more	☐	☐
7–9	☐	☐
5–6	☐	☐
3–4	☐	☐
1–2	☐	☐
Less than 1	☐	☐

Alcohol units

In order to compare different types of alcohol, we ask for the number of alcohol units (= 1.5 cl of pure alcohol). This means the following in practice:

1 glass (1/3 litre) of beer	= 1 unit
1 wine glass of red or white wine	= 1 unit
1 wine glass of sherry or other fortified wine	= 1 unit
1 brandy glass of spirits or liqueur	= 1 unit
1 bottle of alcopop/cider	= 1 unit

63. Have you experienced any of the following during the last 3 years:

	No	Yes
Being hit, kicked or attacked physically in any other way?	☐	☐
Being pressured into having sexual intercourse?	☐	☐

64. Have you during the last 18 months:
(Enter a cross in a box for each item.)

	No	Yes
1. Thought yourself that you were too fat? . .	☐	☐
2. Been really afraid of putting on weight or becoming too fat?	☐	☐
3. Heard others say that you were too thin, while you yourself thought that you were too fat?	☐	☐
4. Thought that it was extremely important for your self-image to maintain a particular weight?	☐	☐

65. Have you at some time during the last 18 months ~~or previously in your life -~~ for a period lasting at least 3 months - experienced any of the following situations, and if so, how frequently was this? *(Enter a cross in a box for each item.)*

	At least twice a week	1-4 times a month	Seldom/ never
1. Felt that you were losing control when eating and couldn't stop before you had eaten far too much?	☐	☐	☐
2. Used vomiting to control your weight?	☐	☐	☐
3. Used laxatives to control your weight?	☐	☐	☐
4. Used fasting to control your weight?	☐	☐	☐
5. Used hard physical exercise to control you weight?	☐	☐	☐

66. Have you at some time during the last 18 months gone at least three months without a period in connection with a time when you have been having eating problems? *(without being pregnant)*

☐ No ☐ Yes

67. What is your current weight? ☐☐☐,☐ kg

How tall are you? ☐☐☐ cm

68. Feeling of anxiety and restlessness. *(Enter a cross in a box for the items that apply to you best during the last 6 months.)*

	Never	Seldom	Sometimes	Often	Very often
1. How often do you have problems completing the final aspects of a task when the challenging part is already done?	☐	☐	☐	☐	☐
2. How often do you have problems putting things in the right order when you are involved in tasks that require organisation?	☐	☐	☐	☐	☐
3. When you have a task which requires a great deal of careful preparation, how often do you avoid or put off starting it?	☐	☐	☐	☐	☐
4. How often do you have problems remembering appointments or engagements?	☐	☐	☐	☐	☐
5. When you have to sit still for a long time, how often do you move your hands and feet in an anxious, restless way?	☐	☐	☐	☐	☐
6. How often do you feel hyperactive and obliged to do things, as if you are being driven by an engine?	☐	☐	☐	☐	☐

69. If you have a husband/boyfriend/partner, to what extent do you agree with the following descriptions? *(Enter a cross in a box for each item.)*

	Totally agree	Agree	Slightly agree	Slightly disagree	Disagree	Totally disagree
1. My partner and I have problems in our relationship	☐	☐	☐	☐	☐	☐
2. I am very happy in my relationship	☐	☐	☐	☐	☐	☐
3. My partner is generally understanding	☐	☐	☐	☐	☐	☐
4. I am satisfied with the relationship with my partner	☐	☐	☐	☐	☐	☐
5. We agree on how children should be brought up	☐	☐	☐	☐	☐	☐

70. Have you been bothered during the last 2 weeks by any of the following? *(Enter a cross in a box for each item.)*

	Not bothered	A little bothered	Quite bothered	Very bothered
1. Feeling fearful	☐	☐	☐	☐
2. Nervousness or shakiness inside	☐	☐	☐	☐
3. Feeling hopeless about the future	☐	☐	☐	☐
4. Feeling blue	☐	☐	☐	☐
5. Worrying too much about things	☐	☐	☐	☐
6. Feeling everything is an effort	☐	☐	☐	☐
7. Feeling tense or keyed up	☐	☐	☐	☐
8. Suddenly scared for no reason	☐	☐	☐	☐

71. Have you experienced during the last 18 months any of the following situations? If yes, how painful and difficult was this for you?
(Enter a cross in a box for each item.)

	No	Yes	Not so bad	Painful/ difficult	Very painful/ difficult
1. Have you had problems at work or where you study?	☐	☐	☐	☐	☐
2. Have you had financial problems?	☐	☐	☐	☐	☐
3. Have you been divorced, separated or ended your relationship with your partner?	☐	☐	☐	☐	☐
4. Have you had problems or conflict with family, friends or neighbours? . .	☐	☐	☐	☐	☐
5. Have you been seriously worried that there is something wrong with your child?	☐	☐	☐	☐	☐
6. Have you been seriously ill or injured?	☐	☐	☐	☐	☐
7. Has anyone close to you been seriously ill or injured?	☐	☐	☐	☐	☐
8. Have you been involved in a serious accident, fire or robbery?	☐	☐	☐	☐	☐
9. Have you lost someone close to you?	☐	☐	☐	☐	☐
10. Other	☐	☐	☐	☐	☐

72. In your daily life, how often do you *(Enter a cross in a box for each item.)*

	Seldom/ never	seldom	A few times	Fairly Often	Very often
1. Feel glad about something	☐	☐	☐	☐	☐
2. Feel happy	☐	☐	☐	☐	☐
3. Feel joyful, like everything is going your way, everything is rosy	☐	☐	☐	☐	☐
4. Feel like screaming at somebody or hitting things	☐	☐	☐	☐	☐
5. Feel angry, irritated or annoyed	☐	☐	☐	☐	☐
6. Feel mad at somebody	☐	☐	☐	☐	☐

73. Indicate with a cross whether you agree or disagree with the following statements *(Enter a cross for each statement.)*

	Totally disagree	Disagree	Slightly disagree	Neither agree nor disagree	Slightly agree	Agree	Totally agree
1. My life is largely what I wanted it to be	☐	☐	☐	☐	☐	☐	☐
2. My life is very good	☐	☐	☐	☐	☐	☐	☐
3. I'm satisfied with my life	☐	☐	☐	☐	☐	☐	☐
4. I've achieved so far what's important to me in my life	☐	☐	☐	☐	☐	☐	☐
5. If I could start all over, there is very little I would do differently	☐	☐	☐	☐	☐	☐	☐
6. I really enjoy my work	☐	☐	☐	☐	☐	☐	☐

74. What kind of perception do you have of yourself? *(Enter a cross in a box for each item.)*

	Totally agree	Agree	Disagree	Totally disagree
1. I have a positive attitude towards myself	☐	☐	☐	☐
2. I feel completely useless at times	☐	☐	☐	☐
3. I feel that I don't have much to be proud of	☐	☐	☐	☐
4. I feel that I am a valuable person, as good as anyone else	☐	☐	☐	☐

75. Bringing up your child *(Enter a cross to indicate whether you agree or disagree with the following statements. Enter a cross in a box for each item.)*

	Totally disagree	Partially disagree	Neither/ nor	Partially agree	Totally agree
1. What I do has little influence on my child's behaviour	☐	☐	☐	☐	☐
2. My child is used to getting what he/she wants in any case, so there's no point in even trying to refuse him/her	☐	☐	☐	☐	☐
3. Cuddles and hugs are an important way of showing my child that I love him/her	☐	☐	☐	☐	☐
4. If my child and I have a disagreement it is usually easy to divert him/her . . .	☐	☐	☐	☐	☐
5. My life is mainly becoming controlled by my child	☐	☐	☐	☐	☐
6. I think it is very important for my child to learn to deal with the fact he/she cannot get their own way on everything	☐	☐	☐	☐	☐
7. It is often easier to let my child get his/her own way rather than having to put up with a tantrum	☐	☐	☐	☐	☐
8. Sometimes when I'm tired I let my child get to do things that I usually would not have allowed otherwise	☐	☐	☐	☐	☐
9. It isn't so important what strategies you use to bring up your children; if you love your children they will develop well	☐	☐	☐	☐	☐

Comments

Have you remembered to fill in on page 1 the date on which you completed the questionnaire?

Thank you very much for your help!

Please return the completed questionnaire in the stamped addressed envelope provided to:

Den norske Mor og Barn undersøkelsen
Nasjonalt folkehelseinstitutt
Avd. for medisinsk fødselsregister
Kalfarveien 31
5018 Bergen
