# Supplementary material for: Association between colic and sleep problems in infancy and subsequent development, emotional and behavioral problems: a longitudinal study
Source: BMC Pediatr. 2021 Jan 7;21:23. doi: 10.1186/s12887-020-02483-1 (PMC7788887; doi:10.1186/s12887-020-02483-1)
Supplement: Supplementary file 4 — Additional file 4. [file 12887_2020_2483_MOESM4_ESM.pdf]

den norske **Mor & barn undersøkelsen**

Questionnaire 5 – Your child at 18 months

In this questionnaire we will ask you some questions which you may recognise from previous questionnaires. We do this because we want to continue following your and your child's progress. It will help if you have child's Health card to hand so that you can use the information contained in it.

If you feel that a question is too upsetting or difficult to answer you can skip this question and go on to the next one.

The questionnaire will be processed by a computer. ***It is therefore important that you following these instructions when completing it:***

- Use a blue or black ballpoint pen.
- Put a *cross* in the box that is most relevant like this: ☒
- If you put a cross in the wrong box, correct it by filling in
- Write numbers in the large green *boxes.*

It is important that you only write in the white area of each box like this:

Number: 1 2 3 4 5 6 7 8 9 0

Please do not use this questionnaire. Contact us at morbarn@fhi.no or phone + 47 53 20 40 40 if you need a questionnaire.

- Numbered boxes have two or more squares. When you enter a single-digit number, use the square on the right. *Example: 5 is entered as follows* 5
- Specific information concerning, *for example, medication* should be written on the lines provided. *Write clearly in CAPITAL LETTERS.*
- Remember to fill in the date on which you completed the questionnaire

As soon as you have completed this questionnaire, return it to us in the stamped addressed envelope provided.

Specify the day, month and year when the questionnaire was completed ☐☐ Day ☐☐ Month ☐☐☐☐ Year *(write the year in full, e.g. 2005)*

ABOUT YOUR CHILD

Food and drink

1. What type of milk has your baby been given since he/she was 6 months old?
(You can enter more than one cross.)

Milk type	Child's age in months 6 - 8	9 - 11	12 - 14	15 - 18
1. Breast milk	☐	☐	☐	☐
2. Formula	☐	☐	☐	☐
3. Formula in the case of milk intolerance	☐	☐	☐	☐
4. Whole milk (sweet)	☐	☐	☐	☐
5. Low-fat milk normal (sweet)	☐	☐	☐	☐
6. Extra low-fat milk (sweet)	☐	☐	☐	☐
7. Skimmed milk (sweet)	☐	☐	☐	☐
8. Yogurt with active Lactobacillus, all types	☐	☐	☐	☐
9. Other yogurt	☐	☐	☐	☐
10. Other types of sour milk	☐	☐	☐	☐

Sp.skj. 5 Engelsk 5D MB - 1.000 - 0508 - Bording

2. How often do you give your child the following to drink now that he/she is 18 months old? Select the frequency which is most applicable on average.
(Enter a cross in a box for each item.)

	Never	Less than once a week	1-3 times a week	4-6 times a week	1-2 times in 24 hrs	3-4 times in 24 hrs	5 or more times in 24 hours
1. Breast milk	☐	☐	☐	☐	☐	☐	☐
2. Formula	☐	☐	☐	☐	☐	☐	☐
3. Whole milk	☐	☐	☐	☐	☐	☐	☐
4. Low-fat milk	☐	☐	☐	☐	☐	☐	☐
5. Extra low-fat milk	☐	☐	☐	☐	☐	☐	☐
6. Skimmed milk	☐	☐	☐	☐	☐	☐	☐
7. Yogurt with active Lactobacillus, all types	☐	☐	☐	☐	☐	☐	☐
8. Yogurt, natural	☐	☐	☐	☐	☐	☐	☐
9. Yogurt with fruit	☐	☐	☐	☐	☐	☐	☐
10. Other types of sour milk	☐	☐	☐	☐	☐	☐	☐
11. Tap water	☐	☐	☐	☐	☐	☐	☐
12. Bottled water	☐	☐	☐	☐	☐	☐	☐
13. Cordial, sweetened	☐	☐	☐	☐	☐	☐	☐
14. Cordial, artificially sweetened	☐	☐	☐	☐	☐	☐	☐
15. Juice	☐	☐	☐	☐	☐	☐	☐
16. Fizzy drinks	☐	☐	☐	☐	☐	☐	☐
17. Diet fizzy drinks	☐	☐	☐	☐	☐	☐	☐
18. Other: ____________________	☐	☐	☐	☐	☐	☐	☐

3. Do you give your child the following to drink during the night now that he/she is roughly 18 months old?
(Enter a cross in a box for each item.)

	Never/ seldom	Now and then	Yes, most nights
1. Water	☐	☐	☐
2. Milk or cordial from a cup	☐	☐	☐
3. Milk or cordial from a bottle	☐	☐	☐
4. Breast milk	☐	☐	☐

4. How often do you give your child the following to eat now that he/she is 18 months old? Select the frequency which is most applicable on average.
(Enter a cross in a box for each item.)

	Never	Less than once a week	1-3 times a week	4-6 times a week	1-2 times in 24 hrs	3 or more times in 24 hrs
1. Liver paste sandwich	☐	☐	☐	☐	☐	☐
2. Meat sandwich	☐	☐	☐	☐	☐	☐
3. Fish sandwich (e.g. sardines, mackerel)	☐	☐	☐	☐	☐	☐
4. Cheese sandwich	☐	☐	☐	☐	☐	☐
5. Jam/honey sandwich	☐	☐	☐	☐	☐	☐
6. Sandwich with other filling	☐	☐	☐	☐	☐	☐
7. Baby porridge (instant)	☐	☐	☐	☐	☐	☐
8. Home-made porridge	☐	☐	☐	☐	☐	☐
9. Meat, sausages, meat balls, etc.	☐	☐	☐	☐	☐	☐
10. Fish, fish balls, fish pudding, etc.	☐	☐	☐	☐	☐	☐
11. Pancakes	☐	☐	☐	☐	☐	☐
12. Potatoes	☐	☐	☐	☐	☐	☐
13. Pasta	☐	☐	☐	☐	☐	☐
14. Rice	☐	☐	☐	☐	☐	☐
15. Peas, beans	☐	☐	☐	☐	☐	☐
16. Other cooked vegetables	☐	☐	☐	☐	☐	☐
17. Raw vegetables	☐	☐	☐	☐	☐	☐
18. Fruit	☐	☐	☐	☐	☐	☐
19. Cakes/waffles/biscuits	☐	☐	☐	☐	☐	☐
20. Dessert/ice cream	☐	☐	☐	☐	☐	☐
21. Chocolate	☐	☐	☐	☐	☐	☐
22. Other sweets, jelly beans, other confectionery	☐	☐	☐	☐	☐	☐

5. Do you give your child a home-made dinner or readymade (processed) baby food in a jar?

- ☐ Only home-made
- ☐ Mostly home-made
- ☐ About half and half of each
- ☐ Mostly ready-made
- ☐ Only ready-made

6. How often do you give your child organic food/drink?
(Enter a cross in a box for each item.)

	Never	Sometimes	Often	Almost always
Sweet milk	☐	☐	☐	☐
Buttermilk/yogurt . .	☐	☐	☐	☐
Vegetables/fruit . . .	☐	☐	☐	☐
Porridge/flour/bread	☐	☐	☐	☐
Meat	☐	☐	☐	☐

+

7. Does your child have a reaction to certain foods?

- ☐ No
- ☐ Yes
- ☐ Don’t know

+

8. If yes, what type of food does your child have a reaction to? *(You can enter a cross in more than one box.)*

1. ☐ Whole milk
2. ☐ Skimmed milk/low-fat milk
3. ☐ Cream
4. ☐ Yogurt/buttermilk
5. ☐ Ice cream
6. ☐ Cheese
7. ☐ Raw egg (e.g. egg flip)
8. ☐ Boiled or fried egg
9. ☐ Fish/fish products
10. ☐ Additives
11. ☐ Wheat
12. ☐ Nuts
13. ☐ Soya
14. ☐ Fruit, berries
15. ☐ Vegetables/potatoes
16. ☐ Chocolate
17. ☐ Other sweets
18. ☐ Sugar
19. ☐ Other: ________________________________

9. Are there any foods which you specifically avoid giving your child?

- ☐ No
- ☐ Yes

+

10. If yes, which foods do you try to avoid and how strict are you with your child’s diet?

	Some reduced use compared to normal diet	Not used unmixed but allowed a little bit in different dishes	Use completely avoided (also “hidden” in dishes)
1. Milk	☐	☐	☐
2. Eggs	☐	☐	☐
3. Fish/fish products	☐	☐	☐
4. Meat/meat products	☐	☐	☐
5. Wheat	☐	☐	☐
6. Sugar	☐	☐	☐
7. Other: ____	☐	☐	☐

11. Do you give your child cold liver oil, vitamins, iron or any other dietary supplement?

- ☐ No
- ☐ Yes

+ +

12. If yes, specify which product(s) and how often you give them to your child. How old was your child when you first started giving him/her the product?

	How often do you give it to your child?		How old was your child when you first gave him the product?
	Every day	sometimes	Number of months
1. Cod liver oil	☐	☐	☐☐
2. Biovit	☐	☐	☐☐
3. Sanasol	☐	☐	☐☐
4. Nycoplus Multi-Vitamin mixture for children	☐	☐	☐☐
5. Fluoride tablets	☐	☐	☐☐
6. Iron supplement, specify: ________________________________	☐	☐	☐☐
7. Other dietary supplement, specify:____________________________	☐	☐	☐☐

Growth, health and illness

Consult your child's health card and use the information contained in it to complete the following questions.

13. How many times have you been to the mother and child health centre since his/her birth?

☐ 0 - 4
☐ 5 -10
☐ 11 -15
☐ 16 or more

14. Do you want your child to be given the vaccinations that are recommended for children in Norway?

☐ Yes, all the recommended vaccinations
☐ Yes, some vaccinations
☐ No, no vaccinations

15. Indicate whether your child has had any vaccinations. If yes, how many times, and indicate if there have been any sideeffects requiring a doctor or hospital to be contacted. *(Enter a cross in a box for each item.)*

			If yes, how many times?			Side-effect resulting in extra contact with a doctor?		Side-effect resulting in examination/admission to hospital?	
**Vaccinations**	No	Yes	1	2	3	No	Yes	No	Yes
1. DTP (diphtheria, tetanus, whooping cough)	☐	☐	☐	☐	☐	☐	☐	☐	☐
2. Hib (Haemophilus influenzae type b)	☐	☐	☐	☐	☐	☐	☐	☐	☐
3. Polio	☐	☐	☐	☐	☐	☐	☐	☐	☐
4. MMR (measles, mumps, rubella)	☐	☐	☐	☐	☐	☐	☐	☐	☐
5. DT (diphtheria, tetanus - sometimes given instead of DTP)	☐	☐	☐	☐	☐	☐	☐	☐	☐
6. Hepatitis B	☐	☐	☐	☐	☐	☐	☐	☐	☐
7. BCG (tuberculosis)	☐	☐	☐	☐	☐	☐	☐	☐	☐
8. Pneumococcus (Prevenar)	☐	☐	☐	☐	☐	☐	☐	☐	☐
9. Other vaccination:____________________________	☐	☐	☐	☐	☐	☐	☐	☐	☐

The following questions concern any illnesses or health problems your child has had. We will first ask you about more long-term problems, then about illnesses and problems of a more acute nature.

16. Does your child have or has he/she had any of the following health problems? If yes, has your child been referred for a specialist examination? *(Enter a cross in a box for each item.)*

				If yes, has child been referred? for a specialist examination?	
**Health problem**	No	Yes, has now	Yes, had previously	No	Yes
1. Dislocated hip (hip problem)	☐	☐	☐	☐	☐
2. Reduced hearing	☐	☐	☐	☐	☐
3. Impaired vision	☐	☐	☐	☐	☐

(cont.)

Health problem	No	Yes, has now	Yes, had previously	If yes, has child been referred for specialist examination? No	Yes
4. Delayed motor development (e.g. sits/walks late) . .	☐	☐	☐	☐	☐
5. Too little weight gain.	☐	☐	☐	☐	☐
6. Too much weight gain	☐	☐	☐	☐	☐
7. Abnormal head circumference	☐	☐	☐	☐	☐
8. Heart defect	☐	☐	☐	☐	☐
9. Testicles not descended into scrotum	☐	☐	☐	☐	☐
10. Asthma	☐	☐	☐	☐	☐
11. Atopic eczema (childhood eczema)	☐	☐	☐	☐	☐
12. Urticaria (hives)	☐	☐	☐	☐	☐
13. Food allergy/intolerance	☐	☐	☐	☐	☐
14. Late or abnormal speech development	☐	☐	☐	☐	☐
15. Sleep problems.	☐	☐	☐	☐	☐
16. Behavioural problems.	☐	☐	☐	☐	☐
17. Social problems	☐	☐	☐	☐	☐
18. (Other) malformations: ______________________	☐	☐	☐	☐	☐
19. Other: ___________________________________	☐	☐	☐	☐	☐

17. If a specialist referral was made, what did this examination show?

☐ Everything was fine
☐ Still some doubts/further examinations needed
☐ Has not been for any examination yet

☐ Diagnosis I: ____

☐ Diagnose II: ____

☐ Diagnose III: ____

18. Has your child been treated with a "cushion" for a hip problem?

☐ No

☐ Yes How long? ☐☐ months

19. Has your child had any of the following illnesses/health problems between 6 and 11 months and/or 12 and 18 months? Specify how many times and whether your child has been admitted to hospital for this health problem. *(Enter a cross in a box for each item.)*

Illness/health problem	At 6 –11 months: No	At 6 –11 months: Yes	Number of times	At 12 -18 months: No	At 12 -18 months: Yes	Number of times	Was admitted to hospital for this? No	Yes
1.Common cold	☐	☐	☐☐	☐	☐	☐☐	☐	☐
2. Throat infection with confirmed streptococcal infection	☐	☐	☐☐	☐	☐	☐☐	☐	☐
3. Other type of sore throat	☐	☐	☐☐	☐	☐	☐☐	☐	☐
4. Ear infection	☐	☐	☐☐	☐	☐	☐☐	☐	☐
5. Pseudocroup	☐	☐	☐☐	☐	☐	☐☐	☐	☐
6. Bronchitis/RS virus/pneumonia	☐	☐	☐☐	☐	☐	☐☐	☐	☐
7. Gastric flu/diarrhoea	☐	☐	☐☐	☐	☐	☐☐	☐	☐
8. Urinary tract infection	☐	☐	☐☐	☐	☐	☐☐	☐	☐
9. Conjunctivitis	☐	☐	☐☐	☐	☐	☐☐	☐	☐

(cont.)

Illness/health problem	At 6 –11 months No	At 6 –11 months Yes	Number of times	At 12 -18 months No	At 12 -18 months Yes	Number of times	Was admitted to hospital for this? No	Was admitted to hospital for this? Yes
10. Febrile convulsions								
11. Other convulsions (without any fever)								
12. Chickenpox								
13. Injury or accident								
14. Other: ________________________								

20. Has your child been to see the doctor or to the hospital between 6 and 11 months and/or 12 and 18 months?
If yes, specify how many times. *(Enter a cross in a box for each item.)*

	At 6 – 11 months No	At 6 – 11 months Yes	At 6 – 11 months Number of times	At 12-18 months No	At 12-18 months Yes	At 12-18 months Number of times
GP (excluding mother and baby health centre)						
Casualty doctor						
Private specialist						
Hospital outpatient clinic						
Admitted to hospital.						

**21. Has your child been referred to any of the following services?**	No	Yes
Habilitation service		
Educational psychology service		
Child psychiatric outpatient clinic/department		

22. If your child has been examined at or admitted to hospital, give the name of the hospital:

Hospital name: ____

Hospital name: ____

Hospital name: ____

23. Has your child had any of the following symptoms since the age of 6 months? If yes, at what age? *(Enter a cross in a box for each item.)*

	Had symptoms? No	Had symptoms? Yes	If yes, at what age? 6-8 mth	9-11 mth	12-14 mth	15 mth or more
1. Wheezing/whistling in the chest						
2. Tightness in the chest						
3. Coughing at night						
4. Runny nose without a cold						
5. Constipation						
6. Diarrhoea						
7. Itchy rash that comes and goes						

24. Has your child ever been tested for allergies?

☐ No
☐ Yes

25. If yes, what allergens were tested for and what was the result?
(You can enter a cross in more than one box.)

Test:		Was the test positive? No	Yes	Don't know
1. ☐	Milk	☐	☐	☐
2. ☐	Egg	☐	☐	☐
3. ☐	Fish	☐	☐	☐
4. ☐	Mould	☐	☐	☐
5. ☐	Mites	☐	☐	☐
6. ☐	Animals	☐	☐	☐
7. ☐	Pollen	☐	☐	☐
8. ☐	Other: ________________	☐	☐	☐

26. Have you ever tried any kind of so-called alternative medicine on your child since he/she was 6 months old?

☐ No
☐ Yes ☐☐ times

27. If yes, what kind of alternative medicine?

____

____

____

____

____

28. Has your child received any medication since the age of 6 months? *(This means any type of medication, including natural medicines and herbal remedies)*

☐ No
☐ Yes

29. If yes, give the name of the medication and what age your child was when he took it. *(Include all types of medication, as well as natural medicines)*

Name of medicine *(WRITE IN CAPITALS, e.g. APOCILLIN, PARACET)*	How old was your child when he/she took this medication? 6-8 mth	9-11 mth	12-14 mth	15-18 mth
____________________	☐	☐	☐	☐
____________________	☐	☐	☐	☐
____________________	☐	☐	☐	☐
____________________	☐	☐	☐	☐
____________________	☐	☐	☐	☐

30. What were your child's length, weight and head circumference when he/she was around 8 months, 1 year and the last time they were measured (15–18 months)?
(Refer to your child's health card)

	Date of measurement: Day	Month	Year	Length	Head circumference	Weight
Around 8 mth	☐☐	☐☐	☐☐	☐☐,☐ cm	☐☐,☐ cm	☐☐☐☐☐ g
Around 1 year	☐☐	☐☐	☐☐	☐☐,☐ cm	☐☐,☐ cm	☐☐☐☐☐ g
15 - 18 mth	☐☐	☐☐	☐☐	☐☐,☐ cm		☐☐☐☐☐ g

Development and behaviour

In this section you will find some questions repeated in a different form. However, please answer all the questions as well as you can.

31. Can your child walk unaided? No ☐ Yes ☐

If yes, how old was your child when he/she could first walk unaided? **Number:** ☐☐ months.

ASQ

32. The questions that follow are about your child's development at around the age of 18 months. *(Enter a cross in a box for each item.)*

	Yes	Sometimes	Not yet
1. When you ask him/her, does your child go into another room to find a familiar toy or object? (When you ask, for instance: "Where's your ball?", "Go and get your coat" or "Go and get your blanket")	☐	☐	☐
2. Does your child say eight or more words, in addition to "mamma" and "dadda"?	☐	☐	☐
3. Without showing him/her first, does your child point to the correct picture when you say "Show me the cat" or "Where is the dog"?	☐	☐	☐
4. Does your child move around by walking, rather than by crawling on his/her hands and knees?	☐	☐	☐
5. Can your child walk well and seldom fall?	☐	☐	☐
6. Does your child walk down stairs if you hold onto one of his/her hands?	☐	☐	☐
7. Does your child throw a small ball or toy with a forward arm motion? (If he/she simply drops the ball, enter a cross under "Not yet")	☐	☐	☐
8. Does your child stack a small block or toy on top of another? (For example, small boxes or toys about 3 cm in size)	☐	☐	☐
9. Does your child turn the pages in a book by himself/herself? (He/she may turn over more than one page at a time.)	☐	☐	☐
10. Does your child hug dolls or cuddly toys when playing with them?	☐	☐	☐
11. Does your child try to get your attention show you something by pulling your hand or clothes?	☐	☐	☐
12. Does your child come to you when he/she needs help, such as with opening a box?	☐	☐	☐
13. Does your child copy the activities you do, such as wiping up a spill, sweeping, shaving or combing hair?	☐	☐	☐

33. More about your child's development *(Enter a cross in a box for each item.)*

	Yes, usually	Very seldom	Not yet
1. Does your child use sounds or words together with gestures (e.g. uses sounds when pointing or reaching towards toys or objects)?	☐	☐	☐
2. When you look at a distant object and, surprised and excited, say: "Waoo...what's that?", – does he/she turn his/her head in the same direction as you?	☐	☐	☐
3. When you enthusiastically say: "Where is the ball (or other toy)?", will your child point towards the toy, even if it is more than 1 metre away?	☐	☐	☐
4. Does your child show you a toy by looking at you and holding the toy up towards your face (from a distance just so you can look at it)?	☐	☐	☐

34. How typical is the following behaviour of your child? *(Enter a cross in a box for each item.)*

	Very typical	Quite typical	Neither/ nor	Not so typical	Not typical
1. Your child cries easily	☐	☐	☐	☐	☐
2. Your child is always on the go	☐	☐	☐	☐	☐
3. Your child prefers playing with others rather than alone	☐	☐	☐	☐	☐
4. Your child is off running as soon as he/she wakes up in the morning	☐	☐	☐	☐	☐
5. Your child is very sociable	☐	☐	☐	☐	☐
6. Your child takes a long time to warm to strangers	☐	☐	☐	☐	☐
7. Your child gets upset or sad easily	☐	☐	☐	☐	☐
8. Your child prefers quiet, inactive games to more active ones	☐	☐	☐	☐	☐
9. Your child likes to be with people	☐	☐	☐	☐	☐
10. Your child reacts intensely when upset	☐	☐	☐	☐	☐
11. Your child is friendly towards and trusting of strangers	☐	☐	☐	☐	☐
12. Your child complains that certain garments are too tight	☐	☐	☐	☐	☐
13. Your child becomes distressed by having his/her face or hair washed	☐	☐	☐	☐	☐

35. About your child's behaviour We are asking you about how your child usually is. If something happens seldom (for instance, if you have only seen it one or twice), enter a cross under "No". *(Enter a cross in a box for each item.)*

	Yes	No
1. Is your child interested in different sorts of toys or objects and not for instance mainly in cars or buttons?	☐	☐
2. When your child expresses his/her feelings, for instance by crying or smiling, do you usually understand why your child is laughing or crying?	☐	☐
3. Does your child react in a normal way to sensory stimulation, such as coldness, warmth, light, pain or tickling?	☐	☐
4. Can you easily tell from the face of your child how he/she feels?	☐	☐
5. When your child has been left alone for some time, does he/she try to attract your attention, for instance, by crying or calling?	☐	☐
6. Is your child's behaviour without stereotyped repetitive movements, e.g. banging his/her head against the wall or rocking his/her body back and forth?	☐	☐
7. Does your child like to be cuddled?	☐	☐
8. Does your child ever laugh directly at you or at other people?	☐	☐
9. Does your child react when spoken to, for instance, by looking, listening, smiling, speaking or babbling?	☐	☐
10. Does your child ever try to comfort you if you are sad or hurt?	☐	☐
11. Has your child ever had things that he/she seemed to have to do in a very particular way or order, or rituals that he/she has to have you do?	☐	☐
12. Does your child ever do things to get you to laugh?	☐	☐

36. More about your child's play and behaviour. We are asking you again about how your child usually is. If something seldom happens (for instance, if you have only seen it one or twice), enter a cross under "No". *(Enter a cross in a box for each item.)*

	Yes	No
1. Does your child enjoy being swung, bounced on your knee, etc.?	☐	☐
2. Does your child take an interest in other children?	☐	☐
3. Does your child like climbing on things, such as up stairs?	☐	☐
4. Does your child enjoy playing peek-a-boo/hide-and-seek?	☐	☐
5. Does your child ever pretend, for example, to talk on the phone or take care of dolls, or pretend other things?	☐	☐
6. Does your child ever use his/her index finger to point, to ask for something?	☐	☐
7. Does your child ever use his/her index finger to point, to indicate interest in something?	☐	☐
8. Can your child play properly with small toys (e.g. cars or bricks) without just mouthing, fiddling or dropping them?	☐	☐
9. Does your child ever bring objects over to you to show you something?	☐	☐
10. Does your child look you in the eye for more than a second or two?	☐	☐
11. Does you child ever seem oversensitive to noise (e.g. plugging ears)?	☐	☐
12. Does your child smile in response to your face or your smile?	☐	☐
13. Does your child imitate you (e.g. you make a face - will your child imitate it?)?	☐	☐
14. Does your child respond when you call his/her name?	☐	☐
15. If you point at a toy across the room, does your child look at it?	☐	☐
16. Does your child look at things you are looking at?	☐	☐
17. Does your child make unusual finger movements near his/her face?	☐	☐
18. Does your child try to attract your attention to his/her own activity?	☐	☐
19. Have you every wondered if your child is deaf?	☐	☐
20. Does your child understand what people say?	☐	☐
21. Does your child sometimes stare at nothing or wander with no purpose?	☐	☐
22. Does your child look at your face to check your reaction when faced with something unfamiliar?	☐	☐

CBCL ✓

37. To what extent are the following statements true of your child's behaviour during the last two months? *(Enter a cross in a box for each item.)*

	Not true	Somewhat or sometimes true	Very true or often true
1. Can't concentrate, can't pay attention for long	☐	☐	☐
2. Quickly shifts from one activity to another	☐	☐	☐
3. Can't sit still, restless or hyperactive	☐	☐	☐
4. Gets into everything	☐	☐	☐

(cont.)

	Not true	Somewhat or sometimes true	Very true or often true
5. Is mostly happy and contented	☐	☐	☐
6. Clings to adults or too dependent	☐	☐	☐
7. Gets too upset when separated from parents	☐	☐	☐
8. Gets into many fights	☐	☐	☐
9. Hits others	☐	☐	☐
10. Is defiant	☐	☐	☐
11. Doesn't seem to feel guilty after misbehaving	☐	☐	☐
12. Punishment doesn't change his/her behaviour	☐	☐	☐
13. Doesn't eat well	☐	☐	☐
14. Likes almost every kind of food	☐	☐	☐
15. Resists going to bed at night	☐	☐	☐
16. Doesn't want to sleep alone	☐	☐	☐
17. Afraid to try new things	☐	☐	☐
18. Disturbed by any change in routine	☐	☐	☐
19. Too fearful or anxious	☐	☐	☐

38. How often does your child usually wake during the night?

- ☐ 3 or more times every night
- ☐ Once or twice every night
- ☐ A few times a week
- ☐ Seldom or never

39. How many hours in total does your child sleep in 24hrs?

- ☐ 10 hours or less
- ☐ 11 - 12 hours
- ☐ 13 -14 hours
- ☐ 15 hours or more

40. About your worries *(Enter a cross in a box for each item.)*

	No	Yes	Don't know
1. Are you worried about your child's physical development?	☐	☐	☐
2. Are you worried about your child's behaviour?	☐	☐	☐
3. Are you worried because your child is demanding and difficult to cope with?	☐	☐	☐
4. Are you worried because your child is so uninterested in other children?	☐	☐	☐
5. Have you any other worries with regard to your child's health	☐	☐	Specify______________________________

(Use the last page if you need more space to write)

Your child's daily routine

41. Where has your child been cared for during the day? Enter a cross for the various age groups. *(Enter a cross in a box for each item.)*

	At home with his/her mother his/her father	At home with unqualified childminder	At a childminder's	In a day nursery
1. 0–6 months	☐	☐	☐	☐
2. 7-9 months	☐	☐	☐	☐
3. 10-12 months	☐	☐	☐	☐
4. 13-15 months	☐	☐	☐	☐
5. 16-18 months	☐	☐	☐	☐

42. How many hours a week is your child looked after in the current childcare scheme (other than by his/her mother and father)?

Type text here

☐☐ hours

43. How many children in total are looked after in this childcare scheme (if day-care centre, how many in the department)?

☐☐ children

44. Do you and your child live with your child's father?

- ☐ Yes
- ☐ No

45. If your child does not live with his/her father, how much time does your child spend with him?

- ☐ At least half the time
- ☐ At least once a week
- ☐ At least once a month
- ☐ Less often than once a month
- ☐ Never

46. How many times have you moved house since your child was born?

☐☐ times

47. Roughly how many square metres is the living area where you currently live?

☐☐☐ m^2

48. Are the rooms where your child is heated by electrical underfloor heating?

☐ No ☐ Yes

49. If yes, which rooms? *Enter a cross in more than one box, if appropriate)*

- ☐ Living room
- ☐ Kitchen
- ☐ Child's room
- ☐ Bedroom
- ☐ Hall
- ☐ Bathroom
- ☐ Other rooms

50. Has their been any damage caused by damp, any visible fungal/mould growth or mouldy smell in your home during the last year *(You can enter a cross in more than one box.)*

- ☐ No
- ☐ Yes, damage caused by damp
- ☐ Yes, visible fungal/mould growth
- ☐ Yes, mouldy smell

51. What type of drinking water do you have where you live?

- ☐ Water from a public or private water company
- ☐ Water from your own water supply (e.g. own well)
- ☐ Don't know

52. Do you live close to high-voltage lines?

- ☐ No
- ☐ Yes, closer than 50 metres
- ☐ Yes, 50–100 metres away
- ☐ Yes, but more than 100 metres away

53. Are there pets where your child lives or at the childminder's?

- ☐ No
- ☐ Yes, at home
- ☐ Yes, at the childminder's

54. If yes, what kind of pets?
(You can enter a cross in more than one box.)

- ☐ Dog
- ☐ Cat
- ☐ Guinea pig, rabbit, mouse, rat, etc.
- ☐ Budgie, other type of bird
- ☐ Other type of animal:________________________________

55. Is your child ever present in a room where someone smokes?

- ☐ Yes, every day Number of times per day ☐☐
- ☐ Yes, several times a week
- ☐ Yes, sometimes
- ☐ Don't know
- ☐ No

56. How many months old was your child when he/she got his/her first tooth?

Number of months ☐☐

- ☐ Don't remember

57. How often are your child's teeth brushed?

- ☐ Twice a day or more
- ☐ Once a day
- ☐ sometimes
- ☐ Never

58. Do you use fluoride toothpaste when brushing your child's teeth?

- ☐ No
- ☐ Sometimes
- ☐ Yes, usually

59. How often is your child outside at the moment?

- ☐ Seldom
- ☐ Often, but less than one hour a day on average
- ☐ 1 - 3 hours a day on average
- ☐ More than 3 hours a day

60. How many hours on average does your child sit in front of a TV/video every day?

- ☐ 4 hours
- ☐ 3 hours
- ☐ 1 -2 hours
- ☐ Less than 1 hour
- ☐ Seldom/never

61. Does your child go to or has been to swimming classes for babies?

- ☐ No
- ☐ Yes

If yes, how long has your child been going? ☐☐ months

62. Does your child use a dummy/pacifier now at 18 months?

- ☐ Seldom or never
- ☐ Only when he/she goes to sleep
- ☐ Quite often
- ☐ Most of the time

ABOUT YOURSELF

Health, illness and use of medication

63. What is your civil status at the moment?

- ☐ Married
- ☐ Separated/divorced
- ☐ Cohabiting
- ☐ Widowed
- ☐ Single
- ☐ Other

64. Are you pregnant at the moment?

- ☐ No
- ☐ Yes

If yes, how many weeks? ☐☐

65. Are you suffering from a long-term illness that has started during the last 12 months?

- ☐ No
- ☐ Yes, specify ____________________

66. Have you yourself been admitted to hospital during the last 12 months?

- ☐ No
- ☐ Yes, which hospital? ____________________

67. Are you taking at the moment any cod liver oil, vitamins or other dietary supplements?

- ☐ No
- ☐ Yes, specify

1. ____________________
2. ____________________
3. ____________________
4. ____________________

68. What is your current weight?

☐☐☐,☐ kg

69. Have you during the last 6 months or at any time previously: *(Enter a cross in a box for each item.)*

	Last 6 months			Previously		
	Yes	Perhaps	No	Yes	Perhaps	No
1. Felt yourself that you were too fat?	☐	☐	☐	☐	☐	☐
2. Been really afraid of putting on weight or becoming too fat?	☐	☐	☐	☐	☐	☐
3. Heard others say you were too thin, while you yourself thought that you were too fat?	☐	☐	☐	☐	☐	☐
4. Felt that it was extremely important for your self-image to maintain a particular weight?	☐	☐	☐	☐	☐	☐

70. Have you at some time during the last 6 months or previously in your life - for a period lasting at least 3 months - experienced any of the following situations, and if so, how frequently was this? (Select the period you were affected the most.) *(Enter a cross in a box for each item.)*

	Last 6 months			Previously		
	At least twice a week	1-4 times a mth	Seldom/ never	At least twice a week	1-4 times a mth	Seldom/ never
1. Felt that you were losing control when eating and couldn't stop before you had eaten too much?	☐	☐	☐	☐	☐	☐
2. Used vomiting to control your weight?	☐	☐	☐	☐	☐	☐
3. Used laxatives to control your weight?	☐	☐	☐	☐	☐	☐
4. Used fasting to control your weight?	☐	☐	☐	☐	☐	☐
5. Used hard physical exercise to control your weight?	☐	☐	☐	☐	☐	☐

71. Have you at some time during the last six months or previously in your life gone at least three months without any periods (without you being pregnant or giving birth/breast-feeding) in connection with a period when you had eating problems?

- ☐ No, never
- ☐ Yes, during the last 6 months
- ☐ Yes, previously

72. Have you experienced pain during the last 12 months in any of the following places? *(Enter a cross in a box for each item.)*

	Seldom/never	Slight pain	Some pain	Major pain
1. Stomach	☐	☐	☐	☐
2. Arms/legs	☐	☐	☐	☐
3. Neck/shoulders	☐	☐	☐	☐
4. Head	☐	☐	☐	☐
5. Back	☐	☐	☐	☐
6. Pelvis (pelvic girdle pains)	☐	☐	☐	☐

73. Have you experienced any pain in your back or pelvis during the last 12 months. Enter a cross to indicate how much pain you have felt in different places:

	Some pain	Major pain
1. In the small of the back	☐	☐
2. One of the pelvic//sacroiliac joints at the back	☐	☐
3. Both pelvic/sacroiliac joints at the back	☐	☐
4. Over the coccygeal bone	☐	☐
5. In the buttocks	☐	☐
6. Over the pubic bone	☐	☐
7. Groin	☐	☐
8. Other back pains	☐	☐
9. Other pains	☐	☐

74. Currently, do you wake during the night because of pelvic pain?

☐ No, never
☐ Yes, but seldom
☐ Yes, often

75. Do you have such problems walking at the moment because of pelvic pains that you have to use a stick or crutches?

☐ No, never
☐ Yes, but not every day - the pain varies from day to day
☐ Yes, must use a stick or crutches every day

76. Did you receive any treatment for pelvic pain after your last birth?

☐ No
☐ Yes

77. If yes, what type of treatment did you receive?
(You can enter a cross in more than one box.)

☐ Physiotherapy
☐ Chiropractic
☐ Medication
☐ Other:________________________________

78. Do you have any of the following problems at the moment? *(Enter a cross in a box for each problem.)*

	How often do you have problems?					How much at a time?	
**Problems:**	Never	1–4 times a month	1–6 times a week	Once a day	More than Once a day	Drops	Large amounts
1. Incontinence when coughing, sneezing or laughing	☐	☐	☐	☐	☐	☐	☐
2. Incontinence during physical activity (running/jumping)	☐	☐	☐	☐	☐	☐	☐
3. Incontinence with a strong need to urinate	☐	☐	☐	☐	☐	☐	☐
4. Problems retaining faeces	☐	☐	☐	☐	☐		
5. Problems retaining flatus	☐	☐	☐	☐	☐		

79. Do you regularly take medication? *(This means any type of medication, including natural medicines.)*

☐ No
☐ Yes

80. If yes, give the name of the medicines and how often you take them. *(Include all types of medication, as well as natural medicines.)*

Name of medicine *(e.g. APOCILLIN, PARACET)*	How often do you take them? Every day	Every day for certain periods	Sometimes
______	☐	☐	☐
______	☐	☐	☐
______	☐	☐	☐
______	☐	☐	☐

Finances – lifestyle

81. How much leave did you and the child's father take after the birth? *(Specify either the number of months or weeks.)*

	Months		Weeks
Yourself	☐☐	or	☐☐
Child's father	☐☐	or	☐☐

82. Are you in paid employment?

☐ No
☐ Yes

83. If so, how many hours do your work a week?

☐☐,☐ hours

84. If you are in paid employment, have you taken any time off sick since you went back to work? If yes, specify how many days you were off sick.

	Number of days
☐ No	
☐ Yes, due to own illness.	☐☐☐
☐ Yes, due to your child being ill.	☐☐☐

85. Would your current finances allow you to cope with an unexpected bill of NOK 3,000 for a dental visit or a repair, for instance?

☐ No
☐ Yes
☐ Don't know

86. Have you found it difficult sometimes during the last six months to cope with running expenses for food, transport, rent, etc.?

☐ No, never
☐ Yes, but infrequently
☐ Yes, sometimes
☐ Yes, often

87. How often are you so physically active (during your spare time or at work) that you get out of breath and sweat?

	Spare time	At work
1. Never	☐	☐
2. Less than once a week	☐	☐
3. Once a week	☐	☐
4. Twice a week	☐	☐
5. 3-4 times a week	☐	☐
6. 5 times or more a week . . .	☐	☐

88. How often do you exercise at present? *(Enter a cross in a box for each item.)*

**Activity**	Never	1-3 times a month	Once a week	Twice a week	3 times or more a week
1. Walking	☐	☐	☐	☐	☐
2. Brisk walking	☐	☐	☐	☐	☐
3. Running/jogging/orienteering	☐	☐	☐	☐	☐
4. Cycling	☐	☐	☐	☐	☐
5. Training studio/weight training	☐	☐	☐	☐	☐
6. Aerobics/gymnastics/dance without running and jumping	☐	☐	☐	☐	☐
7. Aerobics/gymnastics/dance with running and jumping	☐	☐	☐	☐	☐
8. Dancing (swing/rock/folk)	☐	☐	☐	☐	☐
9. Skiing	☐	☐	☐	☐	☐
10. Ball sports	☐	☐	☐	☐	☐
11. Swimming	☐	☐	☐	☐	☐
12. Riding	☐	☐	☐	☐	☐
13. other	☐	☐	☐	☐	☐

89. What are your and your partner's smoking habits at home at the moment?

	Yourself	Your partner/ husband
1. Don't smoke	☐	☐
2. Smoke sometimes	☐	☐
3. Smoke every day	☐	☐
4. If every day, number of cigarettes per day	☐☐	☐☐

90. How often do you consume alcohol at the moment?

- ☐ Roughly 6–7 times a week
- ☐ Roughly 4–5 times a week
- ☐ Roughly 2-3 times a week
- ☐ Roughly once a week
- ☐ Roughly 1-3 times a month
- ☐ Less often than once a month
- ☐ Never

91. How many units do you usually drink when you consume alcohol? (Enter a cross for both weekends and weekdays). *(See explanation below.)*

	Weekend	Weekdays
10 or more	☐	☐
7-9	☐	☐
5-6	☐	☐
3-4	☐	☐
1-2	☐	☐
Less than 1	☐	☐

Alcohol units

In order to compare different types of alcohol, we ask for the number of alcohol units (= 1.5 cl of pure alcohol). This means the following in practice:

1 glass (1/3 litre) of beer	= 1 unit
1 wine glass of red or white wine	= 1 unit
1 sherry glass of sherry or other fortified wine	= 1 unit
1 brandy glass of spirits or liqueur	= 1 unit
1 bottle of alcopop/cider	= 1 unit

A little more about yourself and how you are keeping now

92. If you have a husband/boyfriend/partner, to what extent do you agree with the following descriptions? *(Enter a cross in a box for each item.)*

	Totally agree	Agree	Slightly agree	Slightly disagree	Disagree	Totally disagree
1. My husband/partner and I have a close relationship	☐	☐	☐	☐	☐	☐
2. My partner and I have problems in our relationship	☐	☐	☐	☐	☐	☐
3. I am very happy in my relationship	☐	☐	☐	☐	☐	☐
4. My partner is usually understanding	☐	☐	☐	☐	☐	☐
5. I often think about ending our relationship	☐	☐	☐	☐	☐	☐
6. I am satisfied with my relationship with my partner	☐	☐	☐	☐	☐	☐
7. We often disagree about important decisions	☐	☐	☐	☐	☐	☐
8. I have been lucky in my choice of partner	☐	☐	☐	☐	☐	☐
9. We agree on how children should be raised	☐	☐	☐	☐	☐	☐
10. I think my partner is satisfied with our relationship	☐	☐	☐	☐	☐	☐

93. Do you have anyone other than your-spouse/boyfriend/partner whom you can seek advice from in a difficult situation?

- ☐ No
- ☐ Yes, 1 or 2 people
- ☐ Yes, more than 2 people

94. How often do you see or talk on the telephone to your family (apart from your household) or close friends?

- ☐ Once a month or less often
- ☐ 2-8 times a month
- ☐ More than twice a week

95. Do you often feel lonely?

- ☐ Almost never
- ☐ Seldom
- ☐ Sometimes
- ☐ Generally
- ☐ Almost always

96. How accurate are these statements to you? *(Enter a cross in a box for each item.)*

	Not accurate	Slightly accurate	Almost accurate	Totally accurate
1. I always manage to solve difficult problems if I try hard enough	☐	☐	☐	☐
2. If anyone opposes me, I find a way to get what I want	☐	☐	☐	☐
3. I am sure that I can cope with unexpected events	☐	☐	☐	☐
4. I am calm when I encounter difficulties because I trust my ability to cope	☐	☐	☐	☐
5. When I am in a difficult situation, I usually find a solution	☐	☐	☐	☐

97. In your daily life, how often do you *(Enter a cross in a box for each item.)*

	Seldom/ never	Fairly seldom	Sometimes	Often	Very often
1. Feel pleased about something	☐	☐	☐	☐	☐
2. Feel happy	☐	☐	☐	☐	☐
3. Feel joyful, as though everything is going your way	☐	☐	☐	☐	☐
4. Feel that you will scream at someone or hit something.	☐	☐	☐	☐	☐
5. Feel angry, irritated or annoyed	☐	☐	☐	☐	☐
6. Feel mad at somebody	☐	☐	☐	☐	☐

98. How do you feel about yourself? *(Enter a cross in a box for each item.)*

	Totally agree	Agree	Disagree	Totally disagree
1. I have a positive attitude towards myself	☐	☐	☐	☐
2. I feel completely useless at times	☐	☐	☐	☐
3. I feel that I do not have much to be proud of	☐	☐	☐	☐
4. I feel that I'm a valuable person, as good as anyone else	☐	☐	☐	☐

99. Have you been bothered by any of the following feelings during the past 2 weeks? *(Enter a cross in a box for each item.)*

	Not bothered	A little bothered	Quite bothered	Very bothered
1. Feeling fearful	☐	☐	☐	☐
2. Nervousness or shakiness inside	☐	☐	☐	☐
3. feeling hopeless about the future	☐	☐	☐	☐
4. Feeling blue	☐	☐	☐	☐
5. Worrying too much about things	☐	☐	☐	☐
6. Feeling everything is an effort	☐	☐	☐	☐
7. Feeling tense or keyed up	☐	☐	☐	☐
8. Suddenly scared for no reason	☐	☐	☐	☐

100. Have you experienced any of the following situations in the last year (since the previous questionnaire)? If yes, how painful and difficult was this for you? *(Enter a cross in a box for each item.)*

			If yes		
	No	Yes	Not so bad	Painful/ difficult	Very painful/ difficult
1. Have had problems at work or where you study	☐	☐	☐	☐	☐
2. Have had financial problems	☐	☐	☐	☐	☐
3. Have been divorced, separated or ended your relationship with your partner	☐	☐	☐	☐	☐
4. Have had problems or conflicts with your family, friends or neighbours	☐	☐	☐	☐	☐
5. Have been seriously worried that there is something wrong with your child	☐	☐	☐	☐	☐
6. Have been seriously ill or injured (your self)	☐	☐	☐	☐	☐
7. Has anyone close to you been seriously ill or injured	☐	☐	☐	☐	☐
8.Have been involved in a serious accident, fire or robbery	☐	☐	☐	☐	☐
9. Have lost someone close to you	☐	☐	☐	☐	☐
10. Have been pressurized into having sexual intercourse	☐	☐	☐	☐	☐
11. Other	☐	☐	☐	☐	☐

101. How would you rate your quality of life?

- ☐ Very poor
- ☐ Poor
- ☐ Neither poor nor good
- ☐ Good
- ☐ Very good

102. How satisfied are you with your health?

- ☐ Very dissatisfied
- ☐ Dissatisfied
- ☐ Neither satisfied nor dissatisfied
- ☐ Satisfied
- ☐ Very satisfied

103. The following questions ask about how much you have experienced certain things in the last two weeks. *(Enter a cross in a box for each item.)*

	Not at all	A little	A certain amount	A lot/ very	Totally/ extremely
1. To what extent do you feel that (physical) pain prevents you from doing what you need to do?	☐	☐	☐	☐	☐
2. To what extent do you need medical treatment to be able to function in your daily life?	☐	☐	☐	☐	☐
3. How much do you enjoy life?	☐	☐	☐	☐	☐
4. To what extent do you feel your life to be meaningful?	☐	☐	☐	☐	☐
5. How well are you able to concentrate?	☐	☐	☐	☐	☐
6. How safe do you feel in your daily life?	☐	☐	☐	☐	☐
7. How healthy is your physical environment?	☐	☐	☐	☐	☐

104. The following questions ask about how completely you experienced or were able to do certain things in the last two weeks. *(Enter a cross in a box for each item.)*

	Not at all/None	A little	To a certain extent	Mostly Almost	Always
1. Do you have enough energy for everyday life?	☐	☐	☐	☐	☐
2. Are you able to accept your bodily appearance?	☐	☐	☐	☐	☐
3. Have you enough money to meet your needs?	☐	☐	☐	☐	☐
4. How accessible is the information that you need in your day-to-day life?	☐	☐	☐	☐	☐
5. To what extent do you have the opportunity for leisure activities?	☐	☐	☐	☐	☐

105. How well are you able to get around?

- ☐ Very badly
- ☐ Badly
- ☐ Neither well nor badly
- ☐ Well
- ☐ Very well

106. The following questions ask you to say how good or satisfied you have felt about various aspects of your life over the last two weeks. *(Enter a cross in a box for each item.)*

	Very dissatisfied	Dis-satisfied	Neither satisfied nor dissatisfied	Satisfied	Very satisfied
1. How satisfied are you with your sleep?	☐	☐	☐	☐	☐
2. How satisfied are you with your ability to perform your daily living activities?	☐	☐	☐	☐	☐
3. How satisfied are you with your capacity for work?	☐	☐	☐	☐	☐
4. How satisfied are you with yourself?	☐	☐	☐	☐	☐
5. How satisfied are you with your personal relationships?	☐	☐	☐	☐	☐
6. How satisfied are you with your sex life?	☐	☐	☐	☐	☐
7. How satisfied are you with the support you get from your friends?	☐	☐	☐	☐	☐
8. How satisfied are you with the conditions where you live?	☐	☐	☐	☐	☐
9. How satisfied are you with your access to health services?	☐	☐	☐	☐	☐
10. How satisfied are you with your transport?	☐	☐	☐	☐	☐

107. The following question relates to how often you have experienced or had negative feelings during the last two weeks?

	Never	Seldom	Quite often	Very often	Always
How often do you have negative feelings, such as blue mood, despair, anxiety, depression?	☐	☐	☐	☐	☐

COMMENTS:

CHILD'S MEASUREMENTS AND WEIGHT

108. If any of the measurements in Question 30 are missing from the child's health card, can we contact the well baby clinic for them?

☐ No

☐ Yes Name of well baby clinic ____

Post code or district ____

Have you remembered to fill in on page 1 the date on which you completed the questionnaire?

Thank you very much for your help!

Please return the completed questionnaire in the stamped addressed envelope provided to:

Den norske Mor og Barn undersøkelsen
Nasjonalt folkehelseinstitutt
Avd. for medisinsk fødselsregister
Kalfarveien 31
5018 Bergen
